# Revisiting the relationship between stomatal size and speed across species – a meta‐analysis

**DOI:** 10.1111/nph.70842

**Published:** 2025-12-28

**Authors:** Nik Woning, Yazen Al‐Salman, Elias Kaiser, Sarah R. Berman, Oliver Brendel, Francisco Javier Cano, Sebastien Carpentier, Mauro Centritto, Paul L. Drake, Maxime Durand, David Eyland, Peter J. Franks, Theo Gerardin, Oula Ghannoum, Matthew Haworth, Liisa Kübarsepp, Tracy Lawson, Didier Le Thiec, Yong Li, Leo F. M. Marcelis, Giovanni Marino, Lorna McAusland, Christopher D. Muir, Ülo Niinemets, Tiago D. G. Nunes, Michael T. Raissig, Kazuma Sakoda, Daisuke Sugiura, Tiina Tosens, Qiangqiang Zhang, Ningyi Zhang, Silvere Vialet‐Chabrand

**Affiliations:** ^1^ Horticulture and Product Physiology, Plant Sciences Group Wageningen University 6798PB Wageningen the Netherlands; ^2^ Centre for Crop Systems Analysis, Plant Sciences Group Wageningen University 6700AK Wageningen the Netherlands; ^3^ ARC Centre of Excellence for Translational Photosynthesis, Hawkesbury Institute for the Environment Western Sydney University 2751 Penrith NSW Australia; ^4^ Research Institute of Agriculture and Life Sciences Seoul National University 08826 Seoul Republic of Korea; ^5^ INRAE, Lorraine University, AgroParisTech, UMR 1434 Silva F54280 Champenoux, Nancy France; ^6^ Instituto de Ciencias Forestales (ICIFOR‐INIA), CSIC 28040 Madrid Spain; ^7^ Bioversity International, Biodiversity for Food and Agriculture 3001 Leuven Belgium; ^8^ Laboratory of Tropical Crop Improvement, Division of Crop Biotechnics KU Leuven 3001 Heverlee Belgium; ^9^ Institute for Sustainable Plant Protection National Research Council of Italy (CNR‐IPSP) 50019 Florence Italy; ^10^ School of Biological Sciences The University of Western Australia 6009 Crawley WA Australia; ^11^ Natural Capital and Ecosystem Restoration Astron Environmental Services 6004 East Perth WA Australia; ^12^ Faculty of Biological and Environmental Science, Organismal and Evolutionary Biology (OEB), Viikki Plant Science Centre (ViPS) University of Helsinki 00014 Helsinki Finland; ^13^ School of Life and Environmental Sciences The University of Sydney 2006 Sydney NSW Australia; ^14^ ARC Training Centre for Smart and Sustainable Horticulture Hawkesbury Institute for the Environment, Western Sydney University Penrith 2751 NSW Australia; ^15^ Institute of Agricultural and Environmental Sciences Estonian University of Life Sciences 51006 Tartu Estonia; ^16^ School of Life Sciences University of Essex CO4 3SQ Colchester UK; ^17^ Department of Plant Science University of Illinois 61801 Urbana‐Champaign IL USA; ^18^ National Key Laboratory of Crop Genetic Improvement, Ministry of Agriculture Key Laboratory of Crop Ecophysiology and Farming System in the Middle Reaches of the Yangtze River, College of Plant Science and Technology Huazhong Agricultural University 430070 Wuhan Hubei China; ^19^ Division of Plant and Crop Sciences, School of Biosciences University of Nottingham LE12 5RD Sutton Bonington UK; ^20^ Department of Botany University of Wisconsin 53711 Madison WI USA; ^21^ Centre for Organismal Studies Heidelberg Heidelberg University 69120 Heidelberg Germany; ^22^ Institute of Plant Sciences & Oeschger Centre for Climate Change Research University of Bern 3013 Bern Switzerland; ^23^ Space Environment and Energy Laboratories NTT Inc. 180‐0012 Musashino‐shi, Tokyo Japan; ^24^ Graduate School of Bioagricultural Sciences Nagoya University 464‐8601 Nagoya Aichi Japan; ^25^ College of Horticulture Nanjing Agricultural University 210095 Nanjing China

**Keywords:** amphistomaty, leaf anatomy, leaf gas exchange, light response, model, stomatal conductance, stomatal kinetics

## Abstract

- The rate of stomatal opening and closure in response to changes in light affects leaf photosynthesis and water use. However, it is unclear how strongly stomatal size (*SS*) and density (*SD*) influence stomatal conductance (*g*_s_) kinetics, and whether variation arises from methodological differences, guard cell type or degree of amphistomaty.
- We divided published records combining stomatal kinetics and anatomical traits from 89 species into kidney and dumbbell‐shaped guard cells, and evaluated four dynamic *g*_s_ models on them. We derived the time constant for an exponential response of *g*_s_ (*τ*) and the maximum rate of change (*Sl*_max_) as well as the ratio of adaxial/abaxial *SD* (*rSD*).
- We found significant differences in parameter estimation between models. Stomatal anatomical traits and kinetic parameters showed large variation across species. While individual anatomical features (*SS*, *SD*, *rSD* and guard cell types) were weakly correlated with stomatal response speed (*τ* and *Sl*_max_), interactions between these features showed significant effects, demonstrating that kinetic performance arises from synergistic rather than additive anatomical relationships.
- Our results call for the use of our unified modeling approach, challenge the generality of the observation that smaller stomata move faster across species and suggest *rSD* as an understudied driver of stomatal kinetics.

## Introduction

Stomata are small, adjustable pores flanked by two specialized cells (guard cells) that regulate leaf CO_2_ and water vapor exchange. This function is pivotal for plants, as there is a trade‐off between CO_2_ entry into the leaves for photosynthesis and water vapor loss, which drives nutrient uptake and leaf cooling. Stomatal pore aperture is regulated in response to a myriad of extrinsic and intrinsic stimuli, including light intensity, air humidity, CO_2_ concentration and leaf hormone concentrations (Merilo *et al*., 2014; Lawson & Matthews, 2020). In natural dynamic environments, stomatal apertures adjust dynamically to balance the rate of CO_2_ and H_2_O exchange (Kaiser *et al*., 2015, 2018; Vialet‐Chabrand *et al*., 2013; Vialet‐Chabrand *et al*., 2017; Lawson & Vialet‐Chabrand, 2019; Durand *et al*., 2022). However, stomatal kinetics are one order of magnitude slower than those of photosynthetic biochemistry, making stomatal conductance (*g*_s_; mol m^−2^ s^−1^) a major limitation to photosynthesis after sudden increases in light intensity (Chazdon & Pearcy, 1986; Lawson *et al*., 2010; Kaiser *et al*., 2020; Long *et al*., 2022), and causing unnecessary water loss after sudden drops in light intensity (Lawson & Blatt, 2014). Under fluctuating light intensity, slow responses of *g*_s_ can limit photosynthesis (McAusland *et al*., 2016) and impact yield (Roche, 2015; Adachi *et al*., 2019); by contrast, efficient regulation of *g*_s_ can improve water‐use efficiency (Pignon *et al*., 2021; Ozeki *et al*., 2022; Al‐Salman *et al*., 2023), making *g*_s_ and factors underlying its kinetics interesting breeding targets (Fischer & Rebetzke, 2018; Faralli *et al*., 2019).

Stomata that perfectly track fluctuations in light intensity would facilitate high photosynthesis while avoiding unnecessary water loss (Allen & Pearcy, 2000; Lawson *et al*., 2012). However, changes in cell turgor are achieved by water movement between cells and are not instantaneous, requiring energy (i.e. adenosine triphospate, ATP) to transport osmolytes between cellular compartments (Colin & Fricker, 1996; Vialet‐Chabrand *et al*., 2021). Therefore, the stomatal response typically needs at least a few minutes to reach a new steady state. This response is often asymmetric for an increase and a decrease in *g*_s_, leading to very diverse kinetics (Ooba & Takahashi, 2003; McAusland *et al*., 2016). Fast increases coupled with slow decreases in *g*_s_ lessen the diffusional limitation of photosynthesis by maintaining higher *g*_s_ under fluctuating light intensity but result in high transpiration (Ooba & Takahashi, 2003; Vico *et al*., 2011). A combination of slow increase and fast decrease in *g*_s_ is seen as a more conservative strategy that causes high intrinsic water‐use efficiency (*W*_i_ = *A*/*g*_s_), and may be most suited for arid climates. The rapidity of stomatal movements can be explained by biochemical limitations (Hosy *et al*., 2003; Lebaudy *et al*., 2008; Vialet‐Chabrand *et al*., 2017, 2021), anatomical features (Franks & Beerling, 2009) and their potential interaction (Raven, 2014). Leaf anatomical features vary among species, and also respond plastically in newly emerging leaves under environmental changes (Hetherington & Woodward, 2003). Stomatal anatomical traits have often been used to explain differences in stomatal rapidity (Drake *et al*., 2013; Raven, 2014), although the question of which anatomical factors are most important in a range of species remains unresolved.

The maximum physically attainable value of *g*_s_ is determined by stomatal density (*SD*; no. mm^−2^ surface area) and pore aperture, which is associated with the size of the stomatal complex (Hetherington & Woodward, 2003; Franks & Beerling, 2009). *SD* is often greater on the lower (abaxial) than on the upper (adaxial) side of leaves, but the functional relevance of variations in *SD* ratios between the upper and lower leaf side (*rSD*) is not clear (Wall *et al*., 2022). A negative correlation exists between stomatal size (*SS*) and *SD* (Franks & Beerling, 2009), which was thought to be due to geometric constraints and/or functional factors (Haworth *et al*., 2023). However, this explanation may not hold in reality due to the typical fraction of the surface area covered by stomata (Liu *et al*., 2025). [Correction added on 12 January 2026, after first online publication: the preceding sentence has been updated.]. In closely related species, using the maximum rate of *g*_s_ change between steady states as an estimator for rapidity, smaller stomata were associated with faster change in *g*_s_ compared with larger stomata (Drake *et al*., 2013; McAusland *et al*., 2016; Zhang *et al*., 2019). The larger surface area to volume ratio of small stomata was the proposed explanation, as this allows relatively more cellular machinery on the membrane surface per unit volume to pump osmolytes across the membrane (Franks & Beerling, 2009; Drake *et al*., 2013; Raven, 2014). From these observations arose the notion that smaller stomata generally move faster, even though reports to the contrary exist (Elliott‐Kingston *et al*., 2016; McAusland *et al*., 2016; Zhang *et al*., 2022). However, these results can also be explained by other mechanisms such as nonlinear relationships between stomatal aperture, *SD* and *g*_s_, in which small movements by many small stomata can result in large changes in *g*_s_, although individual stomata may not respond faster than larger stomata (Kaiser & Kappen, 2001; Vialet‐Chabrand *et al*., 2016). In addition, stomatal anatomy is not uniform across species, with various combinations of guard and subsidiary cells, potentially impacting the speed of response (Fryns‐Claessens & Van Cotthem, 1973; Gray *et al*., 2020; Nunes *et al*., 2020). The literature commonly distinguishes between kidney‐shaped (K) and dumbbell‐shaped (DB) stomata. DB stomata have a mechanical advantage due to the configuration of their subsidiary cells, which allows for differential displacement of the cells and results in faster stomatal opening compared to K stomata (Franks & Farquhar, 2007; Pichaco *et al*., 2024). Thus, the relationship between SS and speed may vary across species, but this remains to be tested.

Some of the uncertainty over the SS–speed relationship may stem from methodological issues and inconsistencies between studies: (1) different metrics to characterize speed; (2) a variety of models assuming different response shapes that in‐ or exclude an initial lag time; this variety complicates interstudy comparisons and meta‐analyses of derived parameters; and (3) SS is estimated in several ways, such as multiplying guard cell length and width (Franks & Beerling, 2009), or by assuming a generic empirical shape, such as an ellipse in the case of K guard cells (Savvides *et al*., 2012). This suggests a need to test different models and methods to understand how they impact our interpretation, and a reanalysis of published data using a unified model.

Previous studies have examined SS–speed relationships using relatively small datasets with a focus on intraspecific variations (Supporting Information Table S1). Due to the vast diversity within the plant kingdom and the methodological differences previously mentioned, we concluded that a re‐examination was necessary to determine whether the observations are generalizable and whether they hold across groups and the methodology used. We used as much primary experimental data as we could gather from previous studies and developed a uniform procedure to compare them. We gathered 1094 datasets of leaf gas exchange and stomatal anatomy, resulting in a dataset covering 89 species, and combined this into one large, consistent dataset. We then interrogated this dataset on the relationships between stomatal anatomical parameters, the time constant (*τ*) and the maximum rate of change (*Sl*_max_). We had four objectives: (1) to investigate the importance of metrics and model choice on the estimation of stomatal speed, (2) to elucidate the consistency of the effect of SS on the speed of *g*_s_ responses, (3) to identify other anatomical traits that underpin trends in stomatal speed variation (such as *SD* or *rSD*) and (4) to promote the use of a unifying model that provides physiologically meaningful traits to describe stomatal kinetics. We expected to reveal that the SS–speed relationship depends on several other phylogenetic, physiological and morphological factors. Thus, it cannot be generalized as an axiomatic relationship based on updated calculations of stomatal speed parameters from a new model.

## Materials and Methods

A dataset of published morphological data and corresponding *g*_s_ time courses after step in‐ or decreases in light intensity was constructed from 17 publications (Dataset S1), many of which presented data on a large range of species. The prerequisite for inclusion of a study was that data were available as single‐replicate data, meaning *g*_s_ was measured on the same biological replicate as morphological data. The dataset includes plants from various lineages grown under differing conditions. Data resulting from stress treatments were disregarded: If data were supplied from a single body of work that looked into the effect of drought stress, only the control (well‐watered) replicates were used; if two conditions were applied that were both not harmful (e.g. two levels of CO_2_ concentration), all data were used, yet treated separately. In total, stomatal morphology was measured in 82 species and stomatal conductance (*g*_s_) time courses in 84 species. Combining the two datasets, a total of 89 unique species were captured, which means that the two datasets overlap partially, but are not complete subsets of each other (Table S1).

### Morphological data

Data on stomatal anatomy represented 678 observations of 130 genotypes belonging to 82 species, and were composed of stomatal length (*SSl*), stomatal width (*SSw*) and SD. Guard cell type (K or DB) was assigned using information from original articles or other scientific publications. *SSl* was used as a proxy of SS in our analyses, as it is less affected by variable stomatal pore aperture under the specific conditions of sampling than *SSw*. *SSw* and *SSl* were averaged for both sides of the leaf, while *SD* was summed. For *Musa spp*. and *Brachypodium distachyon*, stomatal adaxial density was calculated from published adaxial/abaxial density ratios (Brun, 1961; Raissig *et al*., 2017). Stomatal ratio (*rSD*) was calculated as *SD*_adaxial_/*SD*_abaxial_ and was then divided into four functional groups based on boundary layer (Guilioni *et al*., 2008): [*rSD* = 0] (abaxial stomata only), [0 < *rSD* ≤ 0.5] (more abaxial than adaxial stomata), [0.5 < *rSD* ≤ 1] (similar numbers of ab‐ and adaxial stomata) and [*rSD* > 1] (fewer abaxial than adaxial stomata). This was performed to reinterpret the zero‐inflated nature of *rSD* to categorical data that are suitable for use with regressions.

### Stomatal conductance responses to step changes in light intensity

Stomatal conductance data originating from a given publication, from the same genotype and with the same direction of change in light intensity, are referred to as ‘a set’, analogous to a treatment. A total of 1139 *g*_s_ time courses consisting of 658 light increases and 481 light decreases were collected. These belonged to 130 genotypes of 84 species. Datasets were collected using the following criteria: (1) gas exchange measurements consisted of at least one stepwise change in light intensity, while keeping other factors (e.g. leaf‐to‐air vapor pressure deficit and leaf temperature), as constant as possible, and (2) the measurement lasted long enough for *g*_s_ to reach a steady state. Four methods for extracting parameters from time courses were used, and their performance was evaluated. Parameter output of the best method was used for further analysis.

### Curation of *g*_s_ time courses

The data points of each time course that were used for model fitting were automatically labeled using light intensity data, and assigned a label for opening and closure. These were then plotted for manual review and curated by hand if needed. There are three major reasons to curate such time courses by hand: (1) definition of start and end points per light step. End points were by default either the end of the dataset, or a second switch in light intensity. However, this could include secondary responses after an initial steady state was reached, which were excluded; (2) consistency between response curves from various sources that are using different measurement protocols. All response curves were trimmed to at least 5 min of steady‐state data if Point 1 did not apply; and (3) removing erroneous *g*_s_ values directly after a light intensity change. When the light intensity changes within the cuvette of a gas exchange measuring device, fast changes in air temperature and vapour pressure deficit (VPD) result in a transient instability in water vapor concentrations between sample and reference chamber, leading to strong transient changes in calculated *g*_s_ that are not representative of changes in pore aperture. This can result in erroneous initial values for model fitting. Such values were eliminated from the fitting process up to the first point with the closest value to the steady‐state *g*_s_ value before the light intensity change, and with a subsequent clear response (Busch *et al*., 2024). An example of how data were labelled when problems exist can be found in Fig. S1.

**Fig. 1:**
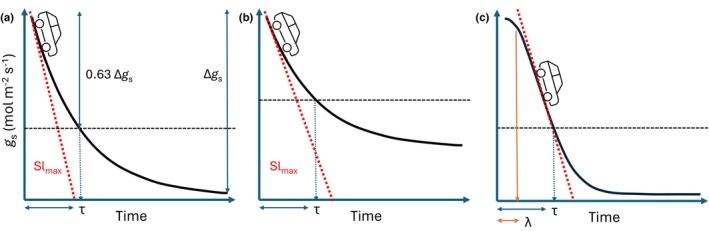
Graphical description of model parameters for an exponential (a, b) and sigmoidal (c) response model of *g*_s_, and analogy of a car descending a mountain road. The black line represents the *g*_s_ kinetic (or road followed by the car); the black dashed line crosses the road when 63% of the variation is achieved. The car is positioned where the maximum speed is reached, corresponding to the estimated maximum slope (Sl_max_). Sl_max_ varies in (a), (b) and (c) despite having the same time constant (*τ*), as the total variation (Δg_s_) changes between a and b and the presence of a lag time (*λ*) in C with the sigmoidal shape. This illustrates the difference in parameter meaning and the importance of the shape of response assumed by the model.

Estimating stomatal speed is often accomplished by deriving parameters from a time course of *g*_s_ after a change in light intensity, assuming that this is equal to the response of a ‘big’ stoma that represents average stomatal behavior (Kaiser, 2009). Two key model parameters are often used to characterize stomatal speed (Fig. 1): *Sl*_max_ and *τ* (tau). While their meanings differ and their interpretations depend on the model, both have been used to describe stomatal speed. To clarify their differences, we use an analogy to describe how fast a car descends a steep mountain road. Typically, average speed, distance and duration of travel are the most intuitive metrics to describe the speed of the car. In this analogy, *Sl*_max_ (mol m^−2^ s^−2^) represents the car's top speed during the journey (Fig. 1a). However, without knowing the road's elevation profile (curve shape), interpreting it can be challenging, as *Sl*_max_ can occur anywhere. If we imagine a car descending the mountain with a smooth transition from a steep decline to a flat valley (exponential decrease), *Sl*_max_ is closely related to acceleration during the initial steep decrease (initial slope) and the difference in elevation between the top and bottom ($\Delta g_{s}$; potential energy; Fig. 1a,b). On the other hand, *τ* (s) represents the time it takes to approach the valley (i.e. duration of travel) and is only related to the relative difference in elevation (e.g. time to reach 63% of the descent, $\Delta g_{s}$). Different elevations between the top and bottom can alter *Sl*_max_ independently of *τ*, if the average speed changes (Fig. 1a,b). Stomatal speed can be defined as the relative rate at which the width of the stomatal aperture changes per second; the rate constant 1/*τ*, which is used to describe a relative change in *g*_s_ per second, comes close to this definition. Additionally, *τ* enables comparison between species with different *g*_s_ variations. Deriving *Sl*_max_ and *τ* assumes a specific shape of the stomatal response, which is typically exponential or sigmoidal. In the case of a sigmoidal shape (Fig. 1c), *τ* and *λ* (the initial lag before a variation in *g*_s_) can be used to calculate the response duration for a relative *g*_s_ decrease. The chosen shape and interpretation of the derived parameter values are therefore crucial for interpreting stomatal speed. The different functions, their possible shapes and how parameters influence this shape are illustrated in Fig. S2 and their equations will be presented later.

#### *t*_63_‐method

Also known as the *t*_50_‐method to estimate the time it takes to reach 50% of the change in *g*_s_ (Grantz & Zeiger, 1986); here, we opted for *t*_63_ to facilitate easy comparison to the other models, as the time constant in exponential models also indicates the time it takes to reach 63% of the magnitude of change. The function is described as:

(Eqn 1) $$g_{s}\left(t_{63}\right)=g_{i}+\left(1-e^{-1}\right)\Delta g_{s}$$

where *g*_i_ denotes initial *g*_s_, and Δ*g*_s_ denotes the difference between final steady‐state *g*_s_ and *g*_i_. A spline with a smoothing factor of 0.005 using the base R function ‘smooth.spline’ was applied to the raw data. From this spline output, initial and final values were captured and *t*_63_ was numerically approached using the spline output.

#### Vico

The method used in Vico *et al*. (2011) describes an exponential curve, a common response in *g*_s_, using the function:

(Eqn 2.1) $$g_{s}=g_{f}+\left(g_{i}-g_{f}\right)e^{-\frac{t}{\tau_{v}}}$$

where *g*_f_ and *g*_i_ denote final and initial steady‐state *g*_s_, respectively, and t denotes time. The time constant *τ*_V_ (s) represents the time it takes to reach 63% of Δ*g*_s_. The maximum slope can be found using:

(Eqn 2.2) $${\mathrm{Sl}}_{\max}=\frac{\left(g_{f}-g_{i}\right)}{\tau_{v}}$$

#### McAusland

This model (McAusland *et al*., 2016) describes a sigmoidal curve, using the following equation:

(Eqn 3.1) $$g_{s}=g_{i}+\left(g_{f}-g_{i}\right)e^{-e^{\left(\frac{\lambda_{M}-t}{\tau_{M}}+1\right)}}$$

where the time constant *τ*_M_ (s) represents the time it takes to reach 37% of Δ*g*_s_ when no lag is considered, and *λ*_M_ is the lag constant (s). t_63_ can be acquired using the following equation:

(Eqn 3.2) $$t_{63}=\lambda_{M}-\left(\ln\left(-\ln\left(1-e^{-1}\right)\right)-1\right)*\tau_{M}$$

Note the dependence of *t*_63_ on *λ*_M_. The maximum slope can be calculated using:

(Eqn 3.3) $${\mathrm{Sl}}_{\max}=\frac{\left(g_{f}-g_{i}\right)}{e\;\tau_{M}}$$

#### CDWeibull

The two‐parameter Cumulative Distribution Weibull (henceforth CDWeibull) describes a stretched exponential and uses the following equation (Weibull, 1939):

(Eqn 4.1) $$g_{s}=g_{f}+\left(g_{i}-g_{f}\right)e^{-{\left(\frac{t}{\tau_{C}}\right)}^{\lambda_{C}}}$$

where the time constant *τ*_C_ represents the time it takes to reach 63% of Δ*g*_s_. The shape constant *λ*_C_ is unitless and determines the shape of the curve. In addition, as *τ*_C_ forms a static point that is independent of *λ*_C_, the time constant of response with and without lag can be compared without recalculating to *t*_63_. This enabled us to use *τ*_C_ as the main estimator of stomatal speed (Fig. S2d).

The maximum slope, or *Sl*_max_ of Eqn 4.1, is described as:

(Eqn 4.2) $${\mathrm{Sl}}_{\max}=\left\{\begin{array}{c} \frac{\left(g_{i}-g_{f}\right)}{\tau_{C}}\;\lambda_{C}=1\\ \frac{-\left(g_{i}-g_{f}\right)\lambda_{C}e^{\frac{\lambda_{C}-1}{\lambda_{C}}}}{{\left(\frac{\lambda_{C}-1}{\lambda_{C}}\right)}^{\frac{1}{\lambda_{C}}}\tau }\;\lambda_{C}>1 \end{array}\right.$$

The shapes of the various models and effects of their parameters can be found in Fig. S2.

### Model fitting

All analyses were performed in R 4.2.1 (R Core Team, 2022). Using Bayesian inference, each model (Eqns 2.1, 3.1, 4.1) was parameterized to fit simultaneously a ‘set’ of at least three *g*_s_ response curves with the same direction that is increase or decrease in light intensity, from the same species and publication. In short, parameter values were altered, then model predictions and observations of *g*_s_ kinetics in response to a change in light intensity were compared, and values minimizing their difference (or likelihood in this case) were selected. Prior distributions and initial start values of the chain can be found in Table S2. The posterior distribution of each parameter was quantified, using four Markov chain Monte Carlo that ran for 1000 sampling iterations (2000 iterations for warming up) with an effective sample size value > 100 (sufficient for describing 95% probability intervals). Target average acceptance probability (Adapt_delta) started on 0.9 and was increased to 0.95 and 0.99 for sets in which divergent transitions where still present. If the sampling still experienced divergent transitions, warm‐up was increased from 2000 to 3000 iterations. Model accuracy was assessed by calculating the root‐mean‐squared error (RMSE). Models were applied to single *g*_s_ time courses using a common estimate for *τ* and *λ* for the whole set and were then scaled using *g*_i_ and *g*_f_. An example is shown in Fig. S3. This process was implemented in the statistical software Stan (http://mc‐stan.org/), using the ‘cmdstanr’ R interface (v.0.9.0; Gabry *et al*., 2025). All chains were checked using the ‘diagnose’ function of ‘cmdstanr’ (Carpenter *et al*., 2017) and were satisfactory for all parameters, except for sets listed in Table S3, which was 4% of all fitted sets. These sets could not converge on parameter values, due to a lack of information in the data (e.g. steady state was not reached, or curves were too different leading to large uncertainty in parameter values), and their output was not used in further analysis.

### Phylogenetic mixed effects Models

Taxonomic names were resolved using the R package ‘taxize’ (v.0.10.0, Chamberlain & Szöcs, 2013). Names provided by the original study authors were queried on 16 August 2025 from the Catalog of Life (Bánki *et al*., 2025). To maximize overlap between the dataset and the GBOTB.extended mega‐tree of seed plants (Zanne *et al*., 2014; Smith & Brown, 2018), the R packages ‘taxonlookup’ (v.1.1.5, Pennell *et al*., 2016) and ‘V.PhyloMaker’ (v.0.1.0, Jin & Qian, 2019) were used. For two species – *Pentarhizidium orientale* and *Shorea leprosula* – taxonomic backbone data were entered manually before using ‘V.PhyloMaker’. Zero‐length branches in the phylogeny were set to the minimum nonzero branch length before analysis.

Bayesian multiresponse mixed effects models with phylogenetically structured random effects were fitted to estimate the explanatory effect of opening and closing speed. The multiresponse approach modeled all response variables (*τ*_open_, *τ*_close_, *Slmax*_open_ and *Slmax*_close_) and their covariance simultaneously. This enabled the estimation of residual covariance among opening and closing rates, potentially reflecting mechanistic and/or correlative relationships between different kinetic responses. Candidate regression models included all possible combinations of *SD* (continuous), *SSl* (continuous), *rSD* (ordered categorical, four levels) and guard cell type, *GC* (unordered categorical, two levels). To account for the ordered nature of *rSD*, polynomial contrasts were applied, allowing the model to capture potential trends across its levels. All continuous variables were natural log‐transformed. All interactions were included in the potential models, and nonhierarchical models were also considered.

To account for missing response variable data, the ‘mi()’ function from the ‘brms’ package was used to integrate over all plausible interpolated values. All combinations of explanatory variables and their two‐way interactions were included in the model set. All models incorporated a random effect of species. Models were fit using a Bayesian framework with Hamiltonian Monte Carlo sampling in the probabilistic programming language Stan via the R package ‘brms’ (v.2.22.0, Bürkner, 2017). ‘CmdStan’ v.2.36.0 and ‘cmdstanr’ v.0.5.3 (Gabry *et al*., 2025) were used for computation. Posterior distributions were sampled using a single chain with 10^3^ sampling iterations following 10^3^ warm‐up iterations.

Model comparison was conducted using the leave‐one‐out cross‐validation information criterion (LOOIC/LOO), calculated with the R package ‘loo’ (v.2.8.0, Vehtari *et al*., 2017). Lower LOO values indicated better out‐of‐sample predictive performance. Additionally, a pseudo‐(Ψ)*R* ^2^ was calculated using the ‘Bayes_R2’ function from ‘brms’ (Gelman *et al*., 2019). For each response variable, the best‐fitting model was selected based on the minimum LOO value. In addition to this best model, more parsimonious models that were treated as equivalent based on LOO values (models with a difference in LOO < 2× the SE in LOO with the best model) and similar Ψ*R* ^2^ were also reported for models with and without phylogenetic structure.

### General statistical analysis

Standard major axis (SMA) analysis was performed using the ‘smatr’ package to test regressions between variables (v.3.4.8; Warton *et al*., 2012). For inference involving *rSD*, a Kruskal–Wallis test was performed with subsequent Dunn test *post hoc* as the data did not satisfy assumptions required for a parametric test, using the base R ‘stats’ and ‘dunn.test’ packages (v.1.3.5; Dinno, 2024). To test whether groups of traits cluster and correlate against other grouped traits, likely creating a more identifiable causal pattern, we conducted a principal component analysis (PCA). Linear correlations for the PCA were calculated using the ‘corrr’ package (v.0.4.4; Kuhn *et al*., 2022). The PCA was constructed using the ‘factoextra’ package (v.1.0.7, Kassambara & Mundt, 2020). PCA stability was checked by means of a LOOCV based on ‘protest’ from the ‘vegan’ package (v.2.6.4; Oksanen *et al*., 2024). The PCA used standard score normalized data. Both SMA and PCAs were performed separately for K and DB guard cells.

## Results

**Fig. 2:**
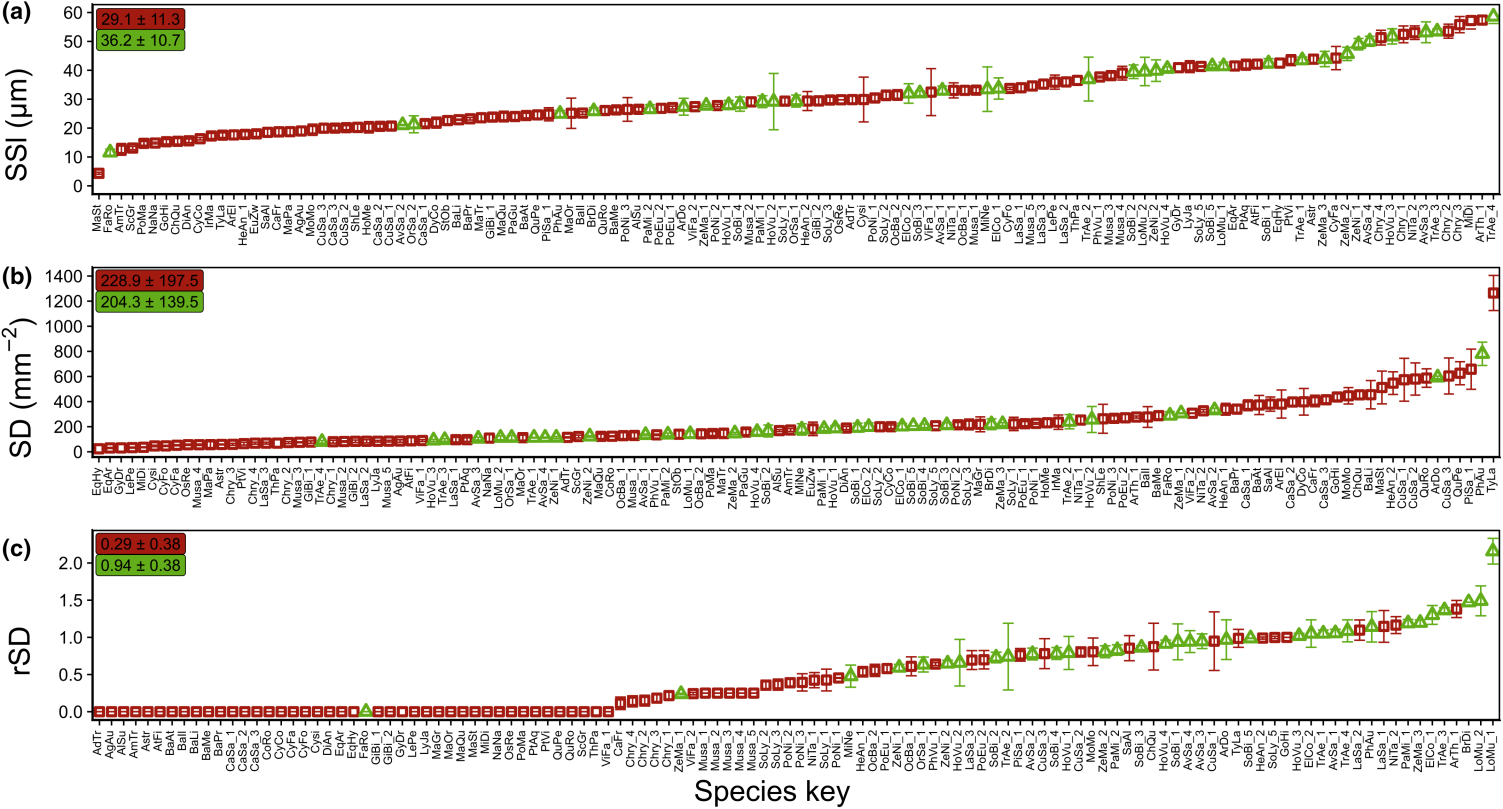
Diversity in stomatal length (μm), stomatal density (mm^−2^) and ratio between adaxial and abaxial stomatal density). Red squares indicate kidney‐shaped stomata, green circles indicate dumbbell‐shaped stomata. The symbols show mean ± SD (error bars) of each species. Text boxes in the upper left corner indicate average ± SD; color indicates respective group. Species keys can be resolved using Supporting Information Dataset S1.

We set out to map the role of stomatal anatomy (size, density and shape) in determining stomatal kinetic responses to a step change in light intensity among plant lineages and crop species. *SSl* varied 13‐fold between 4.3 and 57.4 μm for K and fivefold between 11.6 and 58.6 μm for DB with a mean (± SD) of 29.1 ± 11.3 μm and 36.3 ± 7.2 μm for K and DB, respectively (Fig. 2a). SD in K varied 52‐fold between 24 and 1265 mm^−2^ with a mean of 228.9 ± 197.5 mm^−2^, while in DB, *SD* varied 10‐fold between 80 and 781 mm^−2^, with a mean of 218.6 ± 182.9 mm^−2^ (Fig. 2b). Significant negative correlations between *SD* and *SSl* were found for both groups (*R* ^2^ = 0.33 and 0.49 resp.; *P* < 0.001; Fig. S4). The ratio of adaxial to abaxial *SD* (*rSD*) also showed significant variation from 0 to 2.16 and a mean of 0.29 and 0.94 for K and DB (Fig. 2c). A large fraction of species (29%) only had stomata on the abaxial side, resulting in an *rSD* of 0, and this was more often observed in K (Fig. 2c).

**Fig. 3:**
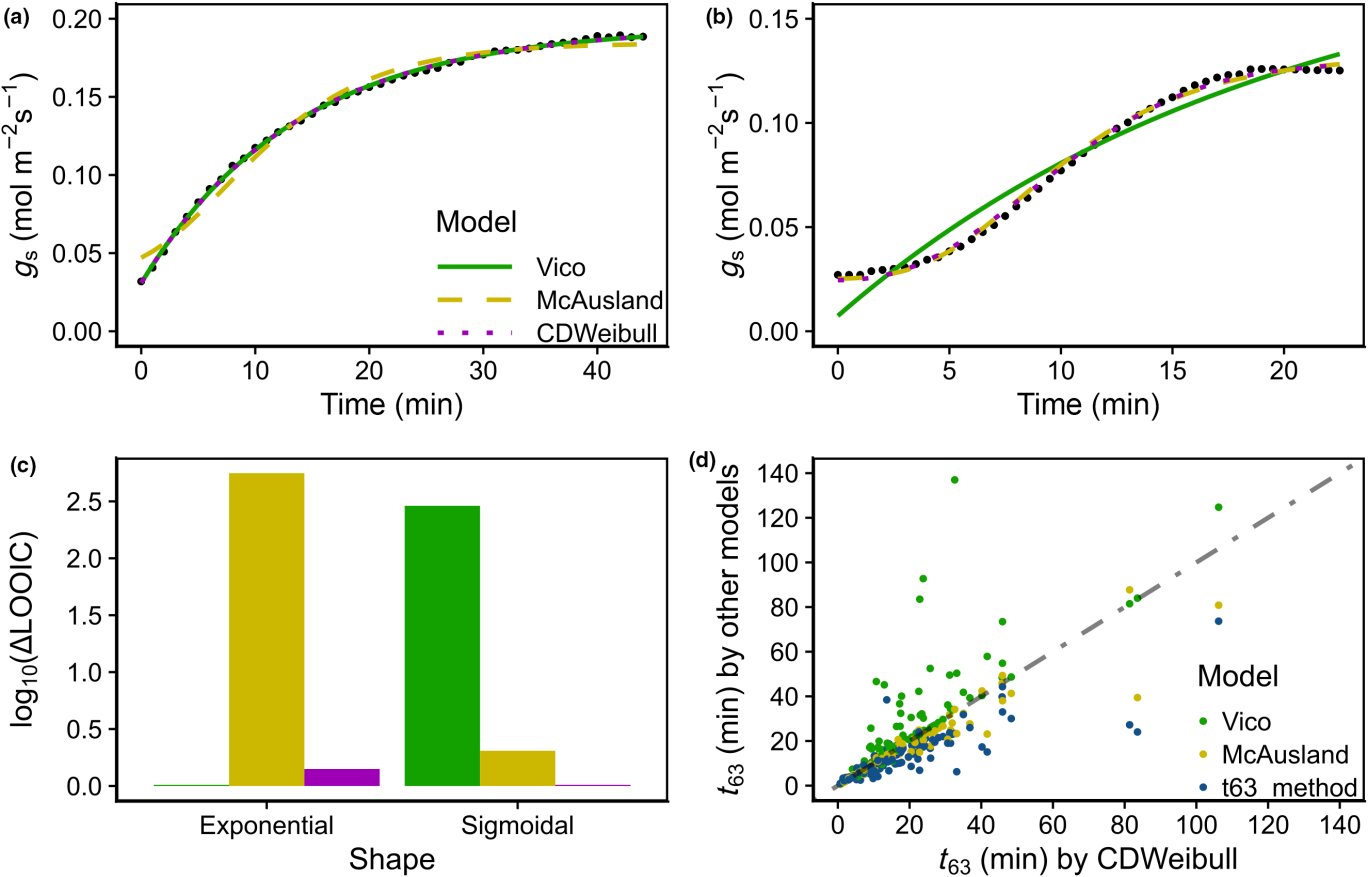
Differences in fit performance of various models. (a, b) Show the fit of the Vico (green solid line; Eqn 2.1), McAusland (yellow dashed line; Eqn 3.1) and CDWeibull models (purple line; Eqn 4.1) on (a) an exponentially shaped *g*_s_ time course of *Pteridium aquilinum* (Replicate 1) from Kübarsepp *et al*. (2020) and (b) a sigmoidally shaped *g*_s_ time course of *Musa* sp. cv Mbwazirume (Replicate 5) from Eyland *et al*. (2021)); measurements are represented by black dots. (c) Shows the difference in the leave‐one‐out cross‐validation information criterion (LOOIC) with the best model for each shape (Vico model for exponential and CDWeibull model for sigmoidal). LOOIC for exponential shape is based fits of *Pteridium aquilinum* (Kübarsepp *et al*., 2020), and LOOIC for sigmoidal shape is based on fits of *Musa* sp. cv Mbwazirume (Eyland *et al*., 2021). (d) shows how the predicted value of *t*_63_ (time to reach 63% of variation in stomatal conductance) of Vico (green dots), McAusland (yellow dots; calculated using *Eqn* *3.2*) and the t63‐method (blue dots) compare with the value of *t*_63_ as predicted by CDWeibull.

We used the compiled *g*_s_ time courses to evaluate the performance of a set of models. The model with the highest accuracy was *CDWeibull* (RMSE: 0.0080 ± 0.0085 mol m^−2^ s^−1^); this model was used for comparison with other models and further analysis (Fig. 3). Compared with *CDWeibull*, *Vico* and *McAusland* had a 15% and 10% larger RMSE, respectively. High RMSE is commonly associated with a poor fit between the model and data. The more accurate estimate of *g*_s_ kinetics using *CDWeibull* stemmed from the difference in shape between models. *CDWeibull* accurately predicted both exponential (Fig. 3a,c) and sigmoidal (Fig. 3b,c) shapes of *g*_s_ kinetics, while *Vico* and *McAusland* showed some limitations. The main problem of the *Vico* and *McAusland* models is the presence or absence of an initial lag after the change in light intensity: We found that while an exponential model (*Vico*) cannot describe the initial lag, the sigmoidal model (*McAusland*) does not fit well on exponential responses without a lag (Fig. S2). Using LOOIC to compare model performance on two representative shapes showed that *CDWeibull* performed similarly well for Vico for exponential shapes, despite having one additional parameter, while outperforming *McAusland* for sigmoidal shapes (Fig. 3c). We found the *t63*‐method to be sensitive to the estimated values of steady‐state *g*_s_, meaning that the estimation of *t*_63_ depended on both the clear presence of steady states and levels of noise in the data. Interestingly, *t*_63_ values estimated with the *t*_63_ method, and to a lesser extent *McAusland*, were underestimated given *CDWeibull* as ground truth, while *Vico* overestimated them (Fig. 3d). The difference between methods led to a maximum difference of *c*. 112 min and a *c*. 590% relative difference in *t*_63_ estimation, altering the ranking of the different species.

**Fig. 4:**
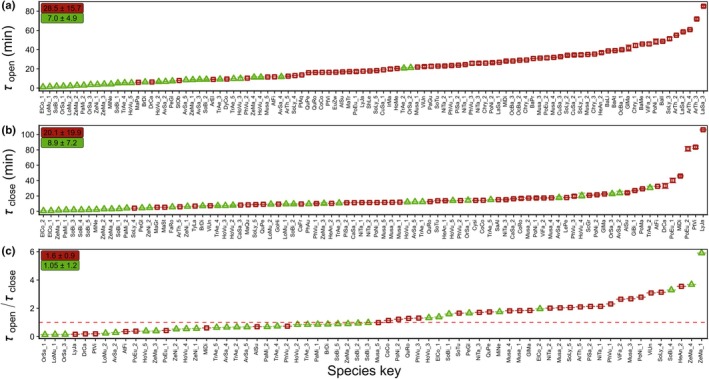
Diversity in the time constant (*τ*) of stomatal opening (a), stomatal closure (b) and their ratio (c), as per the CDWeibull model. Red squares indicate kidney‐shaped stomata, green triangles indicate dumbbell‐shaped stomata. The symbols show mean ± SD (error bars) of each species. Text boxes in the upper left corner indicate average ± SD; color indicates respective group. The red dashed line indicates the 1 line, that is when the time constant (*τ*) for opening is equal to that for closing. Species keys can be resolved using Supporting Information Dataset S1.

There was a large interspecific variation of > 80 min in *τ*_open_. The difference between the fastest (*Eleusine coracana*) and slowest (*Lactuca sativa*) *g*_s_ increase was 80‐fold, with values ranging between 1.1 and 85.2 min (Fig. 4a). Furthermore, we found a > 100‐min difference between the fastest (*E. coracana*) and slowest (*Lygodium japonicum*) *g*_s_ decrease, with values of *τ*_close_ ranging from 0.7 to 106.2 min (Fig. 4b). Mean (± SD) *τ*_open_ in K was 28 ± 16 min, while mean *τ*_close_ was 20 ± 20 min (*P* < 0.05) (Fig. 4a & 4b). Both *τ*_open_ and *τ*_close_ were significantly smaller in DB (*P* < 0.05) but did not significantly differ from one another, with values of 7.0 ± 4.9 min and 8.9 ± 7.2 min. *τ*_open_ and *τ*_close_ were only significantly correlated for DB (*R* ^2^ = 0.28, *P* < 0.01; Fig. S5), and the data showed large variation in the ratio of *τ*_open_ to *τ*_close_ (Fig. 4c), suggesting large diversity in the asymmetry of stomatal responses.

Of 2047 possible model combinations used to characterize the relationships between stomatal anatomical traits and stomatal kinetics, selecting the best models was challenging. This issue stemmed from the intricate relationships among the variables, resulting in multiple equivalent models that exhibited similar predictive performance. We therefore present the best‐performing model based on LOO cross‐validation alongside parsimonious alternatives both with and without phylogenetically structured random effects of species (Table 1).

**Table 1:** Results of phylogenetic multiple regression for various response variables.

Response	Type	SD	SSl	rSD	GC	SD:GC	SSl:GC	rSD:GC	SD:rSD	SSl:rSD	SSl:SD	Lineage	Ψ*R* ^2^	LOO	SE
*τ*_open_	Global	0.018	**0.034**	0.260	0.685	0.675	**0.668**	0.700	**0.266**	**0.264**	0.017	0.762	0.740	79.975	12.209
*τ*_open_	w. Phylogeny	0.018	0.034	0.260	**0.685**	0.675	**0.668**	0.700	0.266	0.264	0.017	**0.762**	0.717	91.194	10.261
*τ*_open_	w.o. Phylogeny	0.018	**0.034**	0.260	0.685	**0.675**	0.668	0.700	0.266	0.264	0.017	0.762	0.698	91.938	11.128
*τ*_close_	Global	**0.073**	0.018	**0.173**	0.299	**0.379**	0.353	**0.364**	**0.219**	0.237	**0.066**	0.608	0.461	114.354	12.402
*τ*_close_	w. Phylogeny	**0.073**	0.018	0.173	0.299	0.379	0.353	0.364	0.219	0.237	0.066	**0.608**	0.606	133.126	10.989
*τ*_close_	w.o. Phylogeny	0.073	**0.018**	0.173	0.299	0.379	**0.353**	**0.364**	0.219	0.237	0.066	0.608	0.453	125.771	11.945
Slmax_open_	Global	**0.089**	**0.013**	0.296	0.555	**0.626**	0.662	**0.580**	**0.326**	**0.369**	0.071	0.675	0.725	88.029	13.929
Slmax_open_	w. Phylogeny	0.089	**0.013**	0.296	0.555	0.626	**0.662**	0.580	0.326	0.369	0.071	**0.675**	0.729	103.864	10.757
Slmax_open_	w.o. Phylogeny	0.089	0.013	0.296	0.555	0.626	**0.662**	**0.580**	0.326	**0.369**	0.071	0.675	0.677	109.184	11.279
Slmax_close_	Global	**0.341**	**0.074**	**0.244**	0.144	0.511	**0.289**	**0.301**	**0.518**	**0.458**	0.197	0.625	0.659	148.386	13.249
Slmax_close_	w. Phylogeny	0.341	0.074	0.244	0.144	0.511	0.289	**0.301**	**0.518**	0.458	0.197	**0.625**	0.690	151.030	8.216
Slmax_close_	w.o. Phylogeny	0.341	0.074	0.244	0.144	**0.511**	0.289	0.301	0.518	**0.458**	0.197	0.625	0.631	160.543	12.490

Response variables are listed in the first column. The second column indicates the model type, which can be: best overall model based on leave‐one‐out (LOO), most parsimonious model with and without phylogeny included based on LOO (leave‐one‐out information criterion (LOOIC)) and Ψ*R* ^2^ (Bayesian *R* ^2^). LOO values indicate the out‐of‐sample predictive performance. Columns three through thirteen report the Ψ*R* ^2^ values from regressions that include only the variable shown in each column. Green highlights/bold text indicate that a specific variable is present in the selected model for each line. In the last three columns, each model type's performance is assessed by its Ψ*R* ^2^ and LOO. For LOO, the SE is provided to compare if models are significantly different. *SD* denotes stomatal density, *SSl* denotes stomatal length, *rSD* denotes the ratio in stomatal densities, *GC* denotes guard cell type, lineage denotes if phylogeny was accounted for in the regression model, and ‘:’ denotes interaction between two factors. All variables were log‐transformed. Regression coefficient estimates can be found in Supporting Information Tables S2, S5–S7.

For *τ*_open_, the best global model (LOO = 79.975 ± 12.209) explained 74.0% of the variance (Ψ*R* ^2^ = 0.740) and included *SSl*, *SSl:GC*, *SD:rSD* and *SSl:rSD*. When phylogenetic relationships were incorporated, model Ψ*R* ^2^ was slightly lower (Ψ*R* ^2^ = 0.717; LOO = 91.194 ± 10.261) and included *GC*, *SSl:GC* and lineage. The most parsimonious model without phylogeny achieved Ψ*R* ^2^ = 0.698 (LOO = 91.338 ± 11.128) using only *SSl* and *SD:GC*.

For *τ*_close_, the best global model explained 46.1% of the variance (Ψ*R* ^2^ = 0.461; LOO = 114.354 ± 12.402) and included *SD*, *rSD*, *SD:GC*, *rSD:GC*, *SD:rSD* and *SSl:SD*. The phylogenetic model showed substantially improved Ψ*R* ^2^ (Ψ*R* ^2^ = 0.608; LOO = 133.126 ± 10.989) and included only *SD* and lineage. The parsimonious model without phylogeny achieved Ψ*R* ^2^ = 0.453 (LOO = 125.771 ± 11.945) using *SSl*, *SSl:GC* and *rSD:GC*.

For *Slmax*_open_, the best global model explained 72.5% of the variance (Ψ*R* ^2^ = 0.725; LOO = 88.029 ± 13.929) and included *SD*, *SSl*, *SD:GC*, *rSD:GC*, *SD:rSD* and *SSl:rSD*. The phylogenetic model showed slightly higher Ψ*R* ^2^ (Ψ*R* ^2^ = 0.729; LOO = 100.864 ± 10.757) and included *SSl*, *SSl:GC* and lineage. The parsimonious model achieved Ψ*R* ^2^ = 0.677 (LOO = 108.184 ± 11.279) using *SSl:GC*, *rSD:GC* and *SSl:rSD*.

For *Slmax*_close_, the global model (Ψ*R* ^2^ = 0.659, LOO = 148.386 ± 13.249) included *SD*, *SSl*, *rSD*, *SSl:GC*, *rSD:GC*, *SD:rSD* and *SSl:rSD*. The phylogenetic model achieved the highest Ψ*R* ^2^ (Ψ*R* ^2^ = 0.690; LOO = 151.030 ± 8.216) and included *rSD:GC*, *SD:rSD* and lineage. The parsimonious model (Ψ*R* ^2^ = 0.631; LOO = 160.543 ± 12.490) required only *SD:GC* and *SSl:rSD*.

Several important patterns emerged from the comprehensive model selection across stomatal kinetic parameters. *SSl* by itself did not explain variations in *τ* or *Slmax* well, but was present in several interactions in the selected models. Interactions that included *rSD* appeared in almost all global models, meaning that interpreting the stomatal speed–size relationship depends on the distribution between abaxial and adaxial sides. The prevalence of interaction terms over main effects highlights the complex, nonadditive nature of how anatomical traits influence stomatal kinetics. The inclusion of phylogenetic relationships revealed variable importance across stomatal functions, with particularly striking improvements for closing speed with *τ*_close_ (Ψ*R* ^2^ increased from 0.461 to 0.608) and *Slmax*_close_ (Ψ*R* ^2^ increased from 0.659 to 0.690). In these phylogenetic models, the lineage effect often replaced multiple anatomical predictors, suggesting that evolutionary history captures additional functional constraints not fully explained by the measured anatomical traits alone. However, the consistently strong performance of parsimonious models demonstrates that key aspects of stomatal kinetic variation can be captured through carefully selected anatomical interactions.

**Fig. 5:**
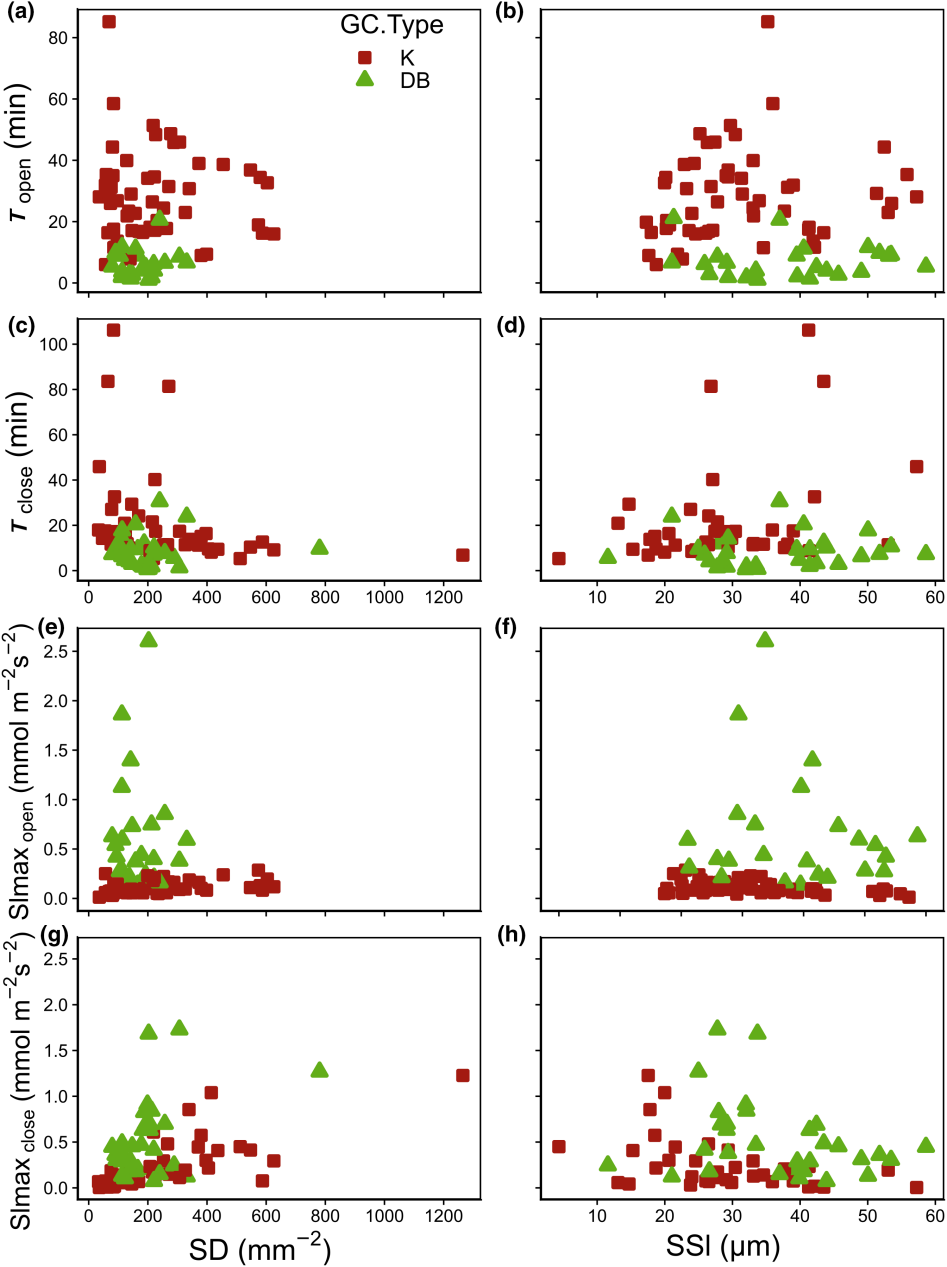
Relation between stomatal speed and anatomy per guard cell (GC) type. Relationships between the time constant of opening (*τ*_open_) and (a) stomatal density (*SD*) and (b) stomatal length (*SSl*). Relationships between *τ*_close_ and (c) *SD* and (d) *SSl*. Relationships between the maximum slope of opening (*Slmax*_open_) and (e) *SD* and (f) *SSl*. Relationships between *Slmax*_close_ and (g) *SD* and (h) *SSl*. Red squares indicate kidney‐shaped stomata, and green triangles indicate dumbbell‐shaped stomata.

**Fig. 6:**
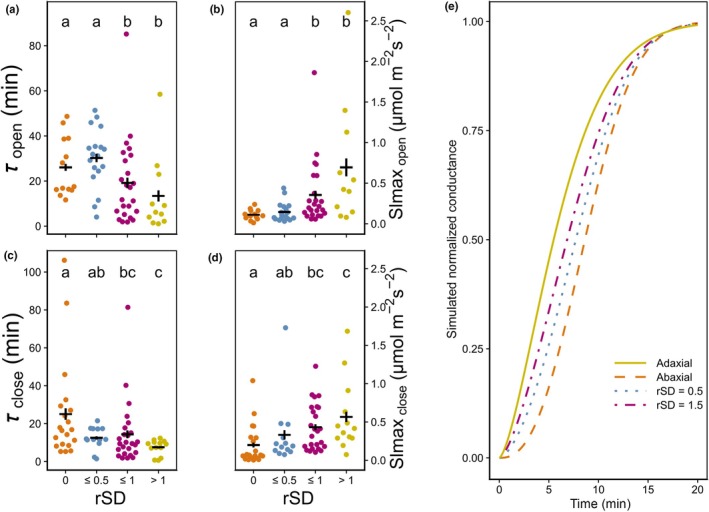
Comparison between model output and anatomy per group. (a) Time constant of opening (*τ*_open_) vs ratio between adaxial and abaxial stomatal densities (*rSD*); (b) maximum slope of opening (*Slmax*_open_) vs *rSD*; (c) *τ*_close_ vs *rSD*; (d) *Slmax*_close_ vs *rSD*; (e) simulation of the role of rSD when stomata on adaxial and abaxial sides show distinct kinetics. CDWeibull (Eqn 4.1) was used to simulate stomatal conductance for adaxial (yellow; *τ* = 7 min & *λ* = 1.5) and abaxial (orange; *τ* = 10 min & *λ* = 2.5) sides independently, and the cumulative kinetics for the leaf are calculated as a weighted average based on rSD (blue, rSD = 0.5; purple, rSD = 1.5). Differences between adaxial and abaxial kinetics are based on Wall *et al*. (2022); Fig. 5a,b). For plots b letters indicate significant differences between groups as per Kruskal–Wallis and Dunn *post hoc*, orange indicates *rSD* of 0 (hypostomatous), blue indicates 0 < *rSD* ≤ 0.5, purple indicates 0.5 < *rSD* ≤ 1, and yellow indicates *rSD* > 1. *rSD* groups are grouped regardless of guard cell type. Black horizontal bars show mean per group; error bars show SE of the mean.

Dynamic parameters such as *τ* were at most weakly correlated with SS and density (Fig. 5) but showed correlation with Δ*g*_s_ (Fig. S6). As parameters were intercorrelated, a PCA was performed to gain insight into their relationship and clustering (Figs S7 and S8). An interesting finding was a relationship between stomatal kinetic parameters and *rSD*. We divided the species here based on *rSD* thresholds (*rSD* = 0, 0 < *rSD* ≤ 0.5, 0.5 < *rSD* ≤ 1, and *rSD* > 1) and found that large *rSD* was related to significantly lower *τ*_open_ (Fig. 6a) and *τ*_close_ (Fig. 6c), as well as higher *Slmax*_open_ and *Slmax*_close_ (Fig. 6b,d, respectively). We then conducted a simulation of leaf *g*_s_ kinetics by separately modeling *g*_s_ time courses on both leaf sides, using the relative contribution of each as a function of *rSD*. Our simulation suggested that, when asymmetry in response is present between leaf sides, variations in *rSD* alter the cumulative rapidity at the leaf level (Fig. 6e).

## Discussion

Many studies have examined the relationship between SS and speed, providing evidence that small stomata respond more rapidly to light intensity changes (Drake *et al*., 2013; Raven, 2014). However, counter examples exist in various species (Elliott‐Kingston *et al*., 2016; McAusland *et al*., 2016; Zhang *et al*., 2022), and this inconsistency may arise from the model used and the interpretation of the metric describing stomatal speed. Applying different models to *g*_s_ time courses led to varying values for fitted parameters on the same dataset, with considerable difference (590%) in estimated *t*_63_ (normalized time constant) and different ranking when comparing species (Fig. 4). To address this issue, we proposed a novel model – CDWeibull – which allowed us to switch between exponential and sigmoidal shapes within the same model, eliminating the need for *a priori* knowledge of the shape of response, and thus facilitating the direct comparison of very diverse responses, providing more accurate fits to the data and more accurate parameter estimates. These improvements were used as a basis for a reanalysis of published results to investigate how anatomical traits influence stomatal speed.

### The stomatal speed–size relationship across species

Our analysis highlighted a large diversity in SS (Fig. 2) and significant variations in the speed of stomatal opening and closing across species (Fig. 4), which we aimed to link. Using the correct methodology is crucial to studying the stomatal speed–size relationship, and here, we list a few points of attention. Correcting gas exchange measurements for time delays between the inlet and outlets of the leaf cuvette (Saathoff & Welles, 2021) can improve estimations of stomatal responses under nonsteady‐state conditions and provide a more accurate time estimation. Methodological differences in estimating SS can play a role in obscuring the relationship, which is why we consistently used *SSl* as a measure of size (de Boer *et al*., 2016). This avoids issues such as artificially low stomatal width, as stomatal imprints often have tightly closed stomata (Rui *et al*., 2024). Beyond gas exchange, incorporating *in situ* microscopy that can probe the response of individual stomata is making it easier to relate size to speed and should be made a common tool to study stomatal kinetics (van den Berg *et al*., 2025).

In general, the relationships between *SSl*, *τ* and *Sl*_max_ were relatively weak across species (Table 1), suggesting that these relationships are not a generic feature as opposed to the relationship between size and density (Fig. S4; Hetherington & Woodward, 2003). However, species‐specific relationships between these parameters can still exist (Drake *et al*., 2013; Raven, 2014), with the limitation that they are sometimes established over limited *SSl* ranges (Fig. S9c). Compared with previous studies, our results stressed that *SSl* alone may not be a good predictor of stomatal speed, but that interaction terms, especially those including guard cell types and the ratio in stomatal densities, could be (Table 1). These two interactions are discussed further later. Including lineage did not significantly improve the model's predictive power but did replace several anatomical predictors. We hypothesized that accounting for guard cell types and the ratio in stomatal densities is already sufficient to distinguish functional groups in the regression analysis and partly overlaps with lineage. It is important to note that *τ* and *Sl*_max_ showed the strongest correlations with Δ*g*_s_ across species and stomatal types (Fig. S6), indicating that stomatal speed is likely also linked to the biochemical regulation of stomatal aperture in steady state. This means that species with large changes in *g*_s_ in response to light intensity are more likely to have fast stomatal responses (Vialet‐Chabrand *et al*., 2016). The SS–speed relationship may hold partially at the species level, but it is too simplistic to account for the complex interaction driving the rapidity of the *g*_s_ response across species.

### The stomatal speed–size relationship for different stomatal types

All selected models relating *τ* and *Sl*_max_ with anatomical traits included *SSl* alone and/or interaction with the stomatal type containing it, which supported the idea that SS influences the rate of change in *g*_s_. Previous publications hypothesized that small stomata were faster due to their larger surface area for osmolyte exchange and lower rates of ion movement to induce guard cell turgor and aperture change (Lawson & Matthews, 2020). Our results suggest that anatomical features such as the structure of the stomatal complex (Franks & Farquhar, 2007; Gray *et al*., 2020; Durney *et al*., 2023) and mechanical constraints (Raven, 2014; Carter *et al*., 2017; Woolfenden *et al*., 2017) that are dependent on the stomatal types play an important role alongside the biochemistry, as the speed depended also on the magnitude of the response (Hosy *et al*., 2003; Lebaudy *et al*., 2008; Ozeki *et al*., 2022). DB stomata generally showed the fastest opening and closing (Fig. 4) as observed in previous publications (Dow & Bergmann, 2014; Nunes *et al*., 2020, 2023). Subsidiary cells in DB stomata are an important part of the stomatal complex, as they provide a mechanical advantage (Franks & Farquhar, 2007; Gray *et al*., 2020; Pichaco *et al*., 2024) and facilitate ion and osmolyte movement to the guard cells, thus accelerating turgor changes (Hosy *et al*., 2003; Lebaudy *et al*., 2008). The size, dimensions and number of subsidiary cells in grasses vary significantly (Nunes *et al*., 2020), and hence can influence stomatal speed and cloud or reduce any effect of SS. Polyploidy is also common in grasses and is usually associated with larger stomatal cells and with slower stomatal opening (Zhou & Osborne, 2024). These variations are likely important in influencing stomatal speed (Rui *et al*., 2024), although further research is necessary to explore the differences among various morphological classes of stomata. Looking more closely within species, the correlation between *Sl*_max_ and *SSl* was significant and negative in barley (Fig. S9a), suggesting that smaller stomata showed faster maximum rates of change, which is the expected relationship. Surprisingly, we found the opposite relationship in bread wheat (Fig. S9b), perhaps due to a larger interface between guard and subsidiary cells allowing for fast ion buildup, enabling a steep change in pore size and *g*_s_. These conflicting results are also reflected by interaction terms having opposite effects per category (Tables S4–S7), which illustrate that the speed–size relationship is not general but can be species‐dependent, and was maybe obscured by our between‐species comparison. Indeed, a key future research goal can be disentangling the effect of C_4_ vs C_3_ photosynthesis and DB stomata, as the energetic requirements to operate DB stomata are likely different in each. Physiological features can play a key role in differentiating the role of anatomy on stomatal speed, and generalizing a specific anatomical influence on stomatal speed as an expected relationship in leaves overall is not viable (Kimura *et al*., 2020; Schulze *et al*., 2021; Clark *et al*., 2022; Horaruang *et al*., 2022).

### Stomatal speed and amphistomatous leaves

Investigating other anatomical traits revealed a relationship between stomatal speed and interactions containing *rSD* (Figs 6, S7), which has previously been reported by Haworth *et al*. (2018) and confirmed here for a larger number of species. Stomata on each side of a leaf respond independently with different speeds and magnitudes (Turner & Singh, 1984; Pearson *et al*., 1995; Soares *et al*., 2008; Wang *et al*., 2008), partly due to the direction of incoming light inducing differences in intensity between leaf sides (Yera *et al*., 1986; Wall *et al*., 2022). In addition, properties such as epidermal layer thickness can be different for upper and lower leaf surfaces (Driscoll *et al*., 2006; Drake *et al*., 2019), which may affect the degree of the mechanical advantage exerted against guard cells and hence the turgor pressure needed to achieve a change in stomatal aperture (Franks & Farquhar, 2007). Differences in spatial distribution of stomata, for example clustering vs even distribution (Shi *et al*., 2021; Sun *et al*., 2023) between upper and lower leaf sides, can affect the change in turgor pressure for stomata to open or close, and thereby affect the speed of the response (Dow *et al*., 2014; Lehmann & Or, 2015). Having different stomatal ratios implies that the relative contribution of each leaf side to total conductance varies (Guilioni *et al*., 2008) and that differences in *g*_s_ kinetics between sides are differently impacted by boundary layer conductance, which affects the overall response. This might bring other morphological factors that affect boundary layer conductance into influence on stomatal speed, such as the presence and density of trichomes (Amada *et al*., 2017, 2023; Buckley *et al*., 2022). Another possible explanation for the relationship between stomatal speed and ratio (Fig. 6) is that faster stomata are generally found on the adaxial side, as recently found in wheat (Wall *et al*., 2022), and, with large *rSD*, have more weight in the response when performing leaf gas exchange measurements, especially when the adaxial side of the leaf is exposed to light (Fig. 6e). As a result, a leaf with higher *rSD* may show faster responses to an increase in light intensity (especially when this is quantified using common leaf gas exchange cuvettes), contributing to an overall faster response (Fig. 6a). Differences in *rSD* between species can change the degree of association between SS (and anatomy overall) on speed in different species creating species‐specific associations (Fig. S9). This advocates for measuring and modeling gas exchange kinetics independently for each leaf side to further our knowledge of leaf gas exchange.

### Understanding the significance of asymmetry in stomatal speed

Although the diversity in time constants and their asymmetry (Fig. 4c) can be attributed to several traits, a question remains about their significance for plants in fluctuating environments. Stomatal behavior can strongly impact water and light use efficiencies, and different ratios of time constants for opening and closing can thus contribute to carbon gain or water‐saving strategies, respectively (Ooba & Takahashi, 2003; Vico *et al*., 2011; McAusland *et al*., 2016). The coordination between speed and magnitude of stomatal responses (Fig. S6c) supports these strategies; for example, a combination of a fast and steep response removes the stomatal limitation of photosynthesis efficiently, but at the expense of high water loss. Muir (2018) suggested that amphistomatous species prevail in high‐light habitats as an adaptation to maximize photosynthesis by increasing CO_2_ diffusion (Jones, 1985). Amphistomaty effectively halves the CO_2_ diffusion path length and boundary layer resistance by doubling boundary layer conductance. Here, we could add that in amphistomatous species, there is also a tendency for faster stomatal responses with different degrees of asymmetry that also contribute to reducing diffusive limitations to nonsteady‐state photosynthesis. However, amphistomatous leaves require significant alterations in stomatal anatomy and the underlying architecture of veins and mesophyll that might require more structural investment (Watts *et al*., 2024) to promote efficient water transport (Rockwell *et al*., 2014; Buckley *et al*., 2015, 2017; Sack *et al*., 2015; Scoffoni, 2015; Onoda *et al*., 2017; Earles *et al*., 2019). Indeed, such trade‐offs would need to be considered when breeding for faster stomata in crops, and hence, experiments screening for stomatal speed need to consider which specific parameter related to speed is most suitable for the specific breeding target and environment. Hence, we encourage recent advances that separate the measurement of *g*_s_ and its responses to the adaxial and abaxial sides, and establish specific relationships relating to size and speed that are side‐specific (Wall *et al*., 2022).

### Conclusions

Our study re‐evaluates the relationship between leaf anatomical traits and stomatal speed, highlighting the importance of model choice and using a novel modeling approach (CDWeibull) to better capture the diversity of stomatal responses across species and stomatal types. While previous studies have proposed a general link between smaller SS and faster responses that span lineages, our results demonstrate that this relationship is not universal. Instead, stomatal speed appears to be context‐dependent, shaped by species‐specific traits, stomatal type and broader anatomical and physiological features. The influence of amphistomaty on response speed underscores the importance of considering both leaf surfaces in physiological measurements. Furthermore, the coordination between stomatal speed, magnitude of conductance change (Δ*g*_s_) and anatomical traits such as SD and *rSD* reveals complex trade‐offs that may underlie adaptive strategies in fluctuating environments. These findings support a trait‐based approach to assessing stomatal kinetics, which has implications for understanding and breeding crops for higher water‐use efficiency and photosynthesis.

## Competing interests

None declared.

## Author contributions

SV‐C and EK designed the original research idea. NW and SV‐C performed the data analysis, with the help of CDM for the phylogenetic regression. SV‐C, EK, NW and YA‐S developed the research design, performed the data interpretation and wrote the first draft of the manuscript. SRB, OB, FJC, SC, MC, PLD, MD, DE, PJF, TG, OG, MH, LK, TL, DLT, YL, LFMM, GM, LM, CDM, UN, TDGN, MTR, KS, DS, TT, QZ and NZ contributed to the manuscript by providing data, comments and revisions. NW and YA‐S contributed equally to this work.

## Disclaimer

The New Phytologist Foundation remains neutral with regard to jurisdictional claims in maps and in any institutional affiliations.

## Supporting information


**Dataset S1** The complete dataset, averaged per species, as used in this work.

**Fig. S1** Example of pre‐processing of stomatal conductance (*g*_s_) data.
**Fig. S2** Differences in model shape and parameter interpretation of the four models.
**Fig. S3** Example of how the model was fitted to all individuals in a ‘set’ and individually scaled to initial and final *g*_s_.
**Fig. S4** Relationships between stomatal density (SD; mm^−2^) and stomatal length (SSl; μm) as per SMA‐regression.
**Fig. S5** Relationship between the time constants for opening (*τ*_open_) and closing (*τ*_close_) as per SMA‐regression.
**Fig. S6** Relationships between the difference in initial and final steady‐state stomatal conductance during opening and stomatal kinetic parameters and anatomy.
**Fig. S7** Principal Component Analysis for stomatal opening and stomatal closing in kidney‐shaped stomata.
**Fig. S8** Principal Component Analysis for stomatal opening and stomatal closing in dumbell‐shaped stomata.
**Fig. S9** Species‐specific relationships between stomatal length (SSl) and maximum speed of stomatal opening (Slmax_open_).

**Table S1** Table showing overview of counts/number observations for various items of interest.
**Table S2** Overview of the priors used for the Bayesian curve fitting process.
**Table S3** Sets that were not used due to divergence during the Bayesian model fitting process.
**Table S4** Regression estimates for multiple regression models for *τ*_open_.
**Table S5** Regression estimates for multiple regression model for *τ*_close_.
**Table S6** Regression estimates for multiple regression model for Slmax_open_.
**Table S7** Regression estimates for multiple regression model for Slmax_close_.
Please note: Wiley is not responsible for the content or functionality of any Supporting Information supplied by the authors. Any queries (other than missing material) should be directed to the *New Phytologist* Central Office.

## Data Availability

Data available in Dataset S1.
