# Supplementary material for: Revisiting the relationship between stomatal size and speed across species – a meta‐analysis
Source: New Phytol. 2025 Dec 28;249(5):2338–54. doi: 10.1111/nph.70842 (PMC12873516; doi:10.1111/nph.70842)
Supplement: Supplementary file 2 — Fig. S1 Example of pre‐processing of stomatal conductance (g s) data. Fig. S2 Differences in model shape and parameter interpretation of the four models. Fig. S3 Example of how the model was fitted to all individuals in a ‘set’ and individually scaled to initial and final g s. Fig. S4 Relationships between stomatal density (SD; mm−2) and stomatal length (SSl; μm) as per SMA‐regression. Fig. S5 Relationship between the time constants for opening (τ open) and closing (τ close) as per SMA‐regression. Fig. S6 Relationships between the difference in initial and final steady‐state stomatal conductance during opening and stomatal kinetic parameters and anatomy. Fig. S7 Principal Component Analysis for stomatal opening and stomatal closing in kidney‐shaped stomata. Fig. S8 Principal Component Analysis for stomatal opening and stomatal closing in dumbell‐shaped stomata. Fig. S9 Species‐specific relationships between stomatal length (SSl) and maximum speed of stomatal opening (Slmaxopen). [file NPH-249-2338-s002.docx]

**New Phytologist Supporting Information**

**Revisiting the relationship between stomatal size and speed across species – a meta-analysis**

Nik Woning, Yazen Al-Salman, Elias Kaiser, Sarah R. Berman, Oliver Brendel, Francisco Javier Cano, Sebastien Carpentier, Mauro Centritto, Paul L. Drake, Maxime Durand, David Eyland, Peter J. Franks, Theo Gerardin, Oula Ghannoum, Matthew Haworth, Liisa Kübarsepp, Tracy Lawson, Didier Le Thiec, Yong Li, Leo F. M. Marcelis, Giovanni Marino, Lorna McAusland18, Christopher D. Muir, Ülo Niinemets, Tiago D.G. Nunes, Michael T. Raissig, Kazuma Sakoda, Daisuke Sugiura, Tiina Tosens, Qiangqiang Zhang, Ningyi Zhang, Silvere Vialet-Chabrand

Article acceptance date: 18 November 2025

***Dataset S1.***

The dataset as used in this body of work. The data is averaged per species. Accompanying data includes further: a reference to the work the data originates from, general information on growth conditions, and species keys to resolve various figures in the main manuscript. This dataset is included in a separate file named ‘SupplementrayFile_1.csv’.

***Table S1. Table showing overview of counts/number observations for various items of interest.*** *Genotypes is here to be understood as species from unique authors. Incr. refers to time courses of increasing stomatal conductance, similarly decr. refers to decreasing stomatal conductance.

*SD* refers to stomatal density, *SSl* refers to stomatal length, *rSD* refers to the ratio of adaxial/abaxial *SD.*

|  | **Data** | **Count** |
| --- | --- | --- |
| **General** | Sources | 17 |
|  | Species | 89 |
|  | Genotypes* | 148 |
|  | Species | 86 |
| **Kinetics** | Sets | 131 |
|  | Sets, incr. | 104 |
|  | Sets, decr. | 96 |
|  | Time course | 1139 |
|  | Time courses, incr. | 658 |
|  | Timecourses, decr. | 481 |
|  | Species | 84 |
| **Morphology** | Genotypes* | 130 |
|  | Total observations | 678 |
|  | SD, total | 647 |
|  | SD, genotypes | 119 |
|  | SSl, total | 660 |
|  | SSl, genotypes | 125 |
|  | SSw, total | 349 |
|  | SSw, genotypes | 67 |
|  | rSD, total | 549 |
|  | rSD, genotypes | 67 |

***Table S2.* *Overview of the priors used for the Bayesian curve fitting process.*** Parameter includes all parameters and hyperparameters used. Type indicates whether the parameter prior is either on an individual curve level, shared on group level or ‘set’, or lastly, a common prior used for all sets. Prior column indicates the distributions and limits set for the prior. Hyperparameter column contains hyperparameters used in the parameter if applicable. Description contains additional information pertaining to the parameter. Notation N(x, y) refers to a normal distribution with mean x and standard deviation y.

***Table S3. Sets that were not used due to divergence during the Bayesian model fitting process.***LN and HN refer to low nitrogen and high nitrogen treatment resp.

| Species/ | Publication | Direction | Divergence |
| --- | --- | --- | --- |
| Cultivar |  |  |  |
| *Sorghum bicolor* | McAusland *et al.*, 2016 | opening | 25 |
| *Eleusine coracana* LN | Ozeki *et al.*, 2022 | opening | 67 |
| *Panicum miliaceum* HN | Ozeki *et al.*, 2022 | opening | 93 |
| *Sorghum bicolor* LN | Ozeki *et al.*, 2022 | opening | 13 |
| *Zea nicaraguensis* LN | Ozeki *et al.*, 2022 | opening | 99 |

***Table S4.*** ***Regression estimates for multiple regression models for τ_open_.*** Global indicates the best overall model based on LOO, w. Phylogeny indicates the most parsimonious model with phylogeny included and w.o. Phylogeny indicates without phylogeny, based on LOO (or LOOIC: Leave-One-Out Information Criterion) and ΨR^2^ (Bayesian R^2^). The Phyl.Intercept estimate can be interpreted as the standard deviation around the Intercept estimate caused by species-specific variation. *SD* refers to stomatal density, *SSl* refers to stomatal length, *rSD* refers to the ratio of adaxial/abaxial *SD*, GC_type(K) and GC_type(DB) refer to kidney and dumbbell shaped stomata resp. Results include linear (.L), quadratic (.Q), and cubic (.C) trend components for the categorical variables.

***Table S5.*** ***Regression estimates for multiple regression model for τ_close_.*** Global indicates best overall model based on LOO, w. Phylogeny indicates most parsimonious model with phylogeny includedand w.o. Phylogeny indicates without phylogeny included, based on LOO (or LOOIC: Leave-One-Out Information Criterion) and ΨR^2^ (Bayesian R^2^). The Phyl.Intercept estimate can be interpreted as the standard deviation around the Intercept estimate causes by species-specific variation. *SD* refers to stomatal density, *SSl* refers to stomatal length, *rSD* refers to the ratio of adaxial/abaxial *SD*, GC_type(K) and GC_type(DB) refer to kidney and dumbbell shaped stomata resp. Results include linear (.L), quadratic (.Q), and cubic (.C) trend components for the categorical variables.

***Table S6.*** ***Regression estimates for multiple regression model for Slmax_open_.*** Global indicates best overall model based on LOO, w. Phylogeny indicates most parsimonious model with phylogeny includedand w.o. Phylogeny indicates without phylogeny included, based on LOO (or LOOIC: Leave-One-Out Information Criterion) and ΨR^2^ (Bayesian R^2^). The Phyl.Intercept estimate can be interpreted as the standard deviation around the Intercept estimate causes by species-specific variation. *SD* refers to stomatal density, *SSl* refers to stomatal length, *rSD* refers to the ratio of adaxial/abaxial *SD*, GC_type(K) and GC_type(DB) refer to kidney and dumbbell shaped stomata resp. Results include linear (.L), quadratic (.Q), and cubic (.C) trend components for the categorical variables.

***Table S7.*** ***Regression estimates for multiple regression model for Slmax_close_.*** Global indicates best overall model based on LOO, w. Phylogeny indicates most parsimonious model with phylogeny includedand w.o. Phylogeny indicates without phylogeny included, based on LOO (or LOOIC: Leave-One-Out Information Criterion) and ΨR^2^ (Bayesian R^2^). The Phyl.Intercept estimate can be interpreted as the standard deviation around the Intercept estimate causes by species-specific variation. *SD* refers to stomatal density, *SSl* refers to stomatal length, *rSD* refers to the ratio of adaxial/abaxial *SD*, GC_type(K) and GC_type(DB) refer to kidney and dumbbell shaped stomata resp. Results include linear (.L), quadratic (.Q), and cubic (.C) trend components for the categorical variables.

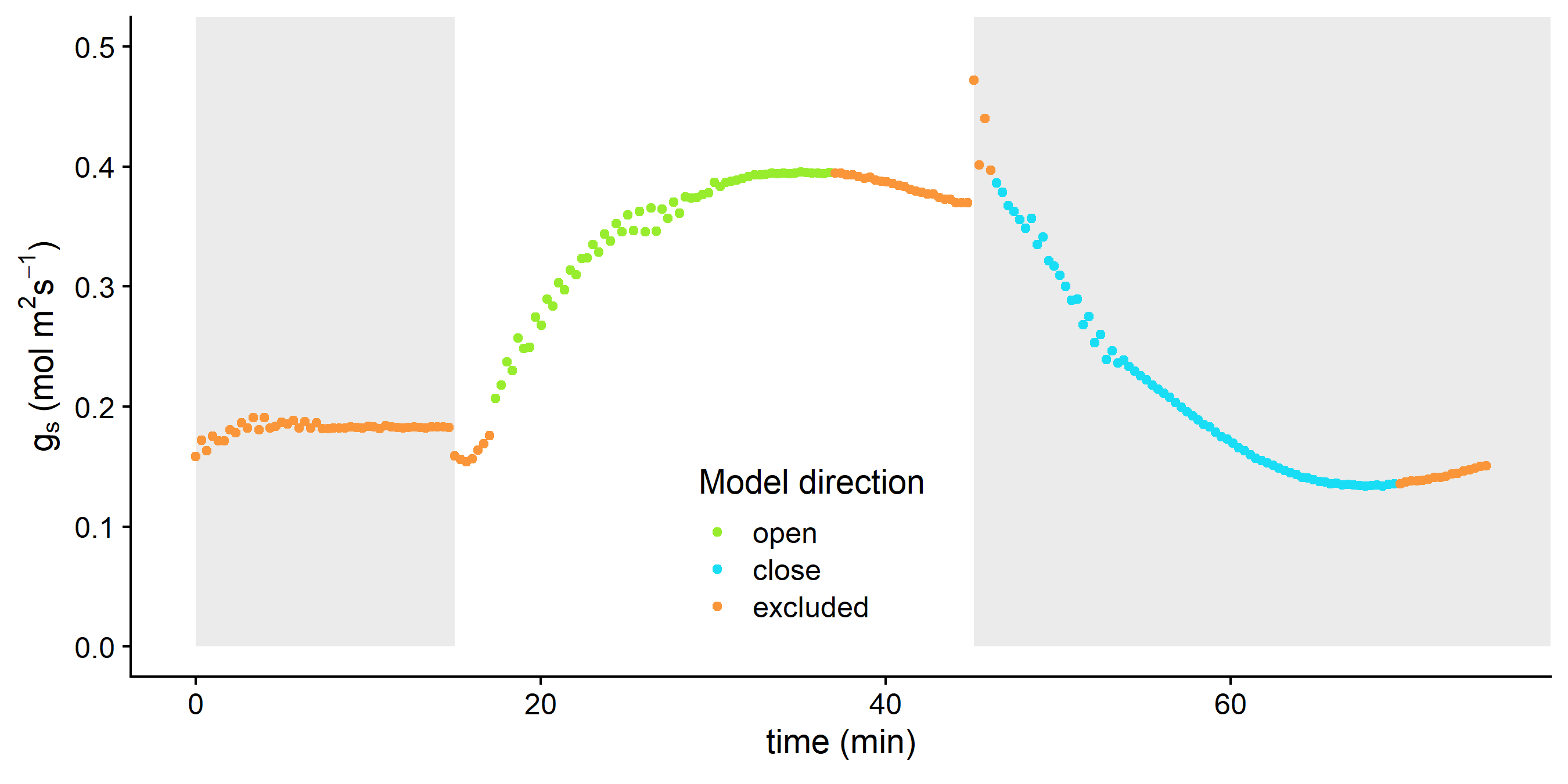


***Figure S1. Example of pre-processing of stomatal conductance (g_s_) data.*** Gray areas denote light intensity (high in white and low in gray). Green dots denote *g_s_* values used for stomatal opening, blue dots denote those used for stomatal closure. The first few data points after a switch to high light intensity, as well as first few data points after a switch from high to low light intensity, are considered ‘erroneous’, and are thus not used. The first value with approximately the same magnitude is picked to start model fitting. However, for the model, time = 0 is set as the moment of light intensity change. In addition, a ‘secondary response’ is visible during the steady state after opening and closing, here a decrease and increase in *g_s_*, respectively. Shown here is a single *g_s_* trace of *Hordeum vulgare* from Vialet-Chabrand et al. (2021).


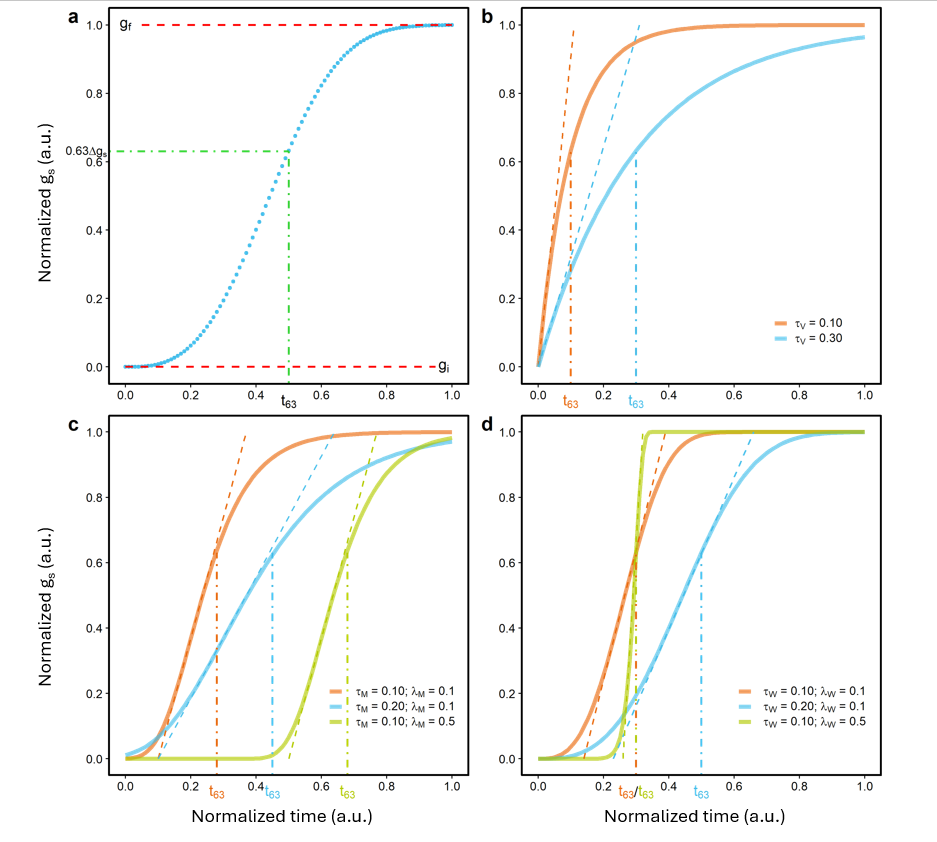


***Figure S2*. *Differences in model shape and parameter interpretation of the four models.*** **(a)** shows the *t_63_*-method, without any underlying model *(eqn. 1).* The blue dotted line indicates hypothetical data values. The red dashed lines indicates steady-state values. The dot-dashed green lines indicate the values of 63% of the magnitude between steady states and associated *t_63_*. **(b)** shows the exponential Vico model (*eqn. 2.1*). **(c)** shows the McAusland model (*eqn. 3.1*). **(d)** shows the CDWeibull model. In **(b, c, d)**, solid lines indicate the various shapes the models take when using the parameter input of the same colour. Dashed lines show maximum slopes associated with these curves, dot-dashed lines indicate *t_63_*. Noteworthy are the following observations: the Vico model is incapable of forming a sigmoidal curve (i.e. it cannot incorporate an initial lag in *g_s_*). McAusland is only capable of forming a sigmoidal curve (no lag is emulated by using very low values for λ, yet it is always present). When using the McAusland model, the addition of lag (λ) shifts *t_63_* with regards to the time constant (*τ*). When using CDWeibull, τ forms a fixed point that equates *t_63_* regardless of the value of λ.


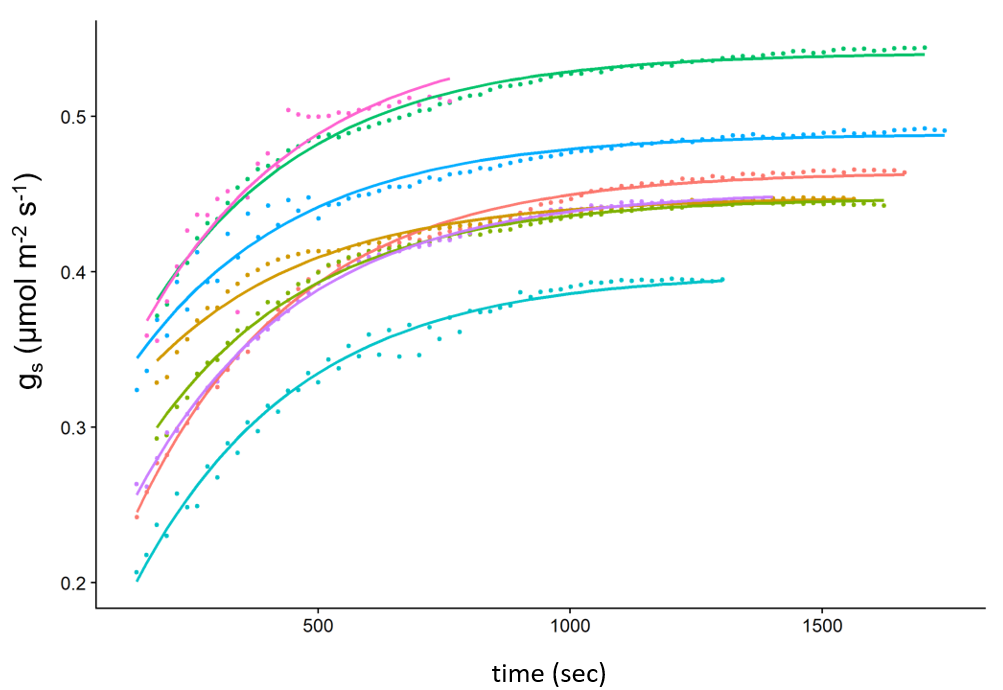
 ***Figure S3. Example of how the model was fitted to all individuals in a ‘set’ with shared τ and λ, and was individually scaled to initial and final g_s_* (stomatal conductance)*.*** Shown are all stomatal opening traces of *Hordeum vulgare* from Vialet-Chabrand et al. (2021), i.e. a ‘set’.


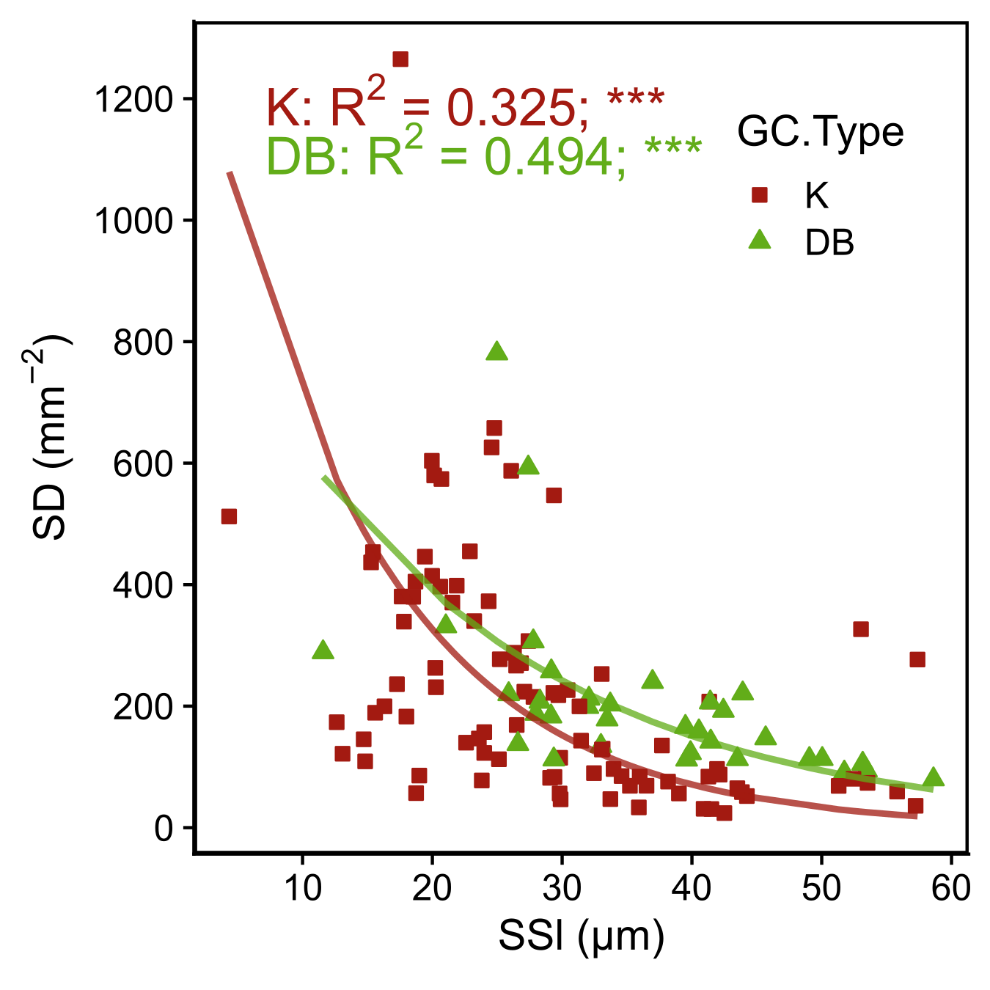


***Figure S4.*** ***Relationships between stomatal density (SD; mm^-2^) and stomatal length (SSl; μm) as per SMA-regression.*** Red squares indicate kidney-shaped stomata (K), green triangles indicate dumbbell-shaped stomata (DB). For the regressions, *SD* data were ln-transformed. Stars indicate the significance level of the regression; *** > 0.001.


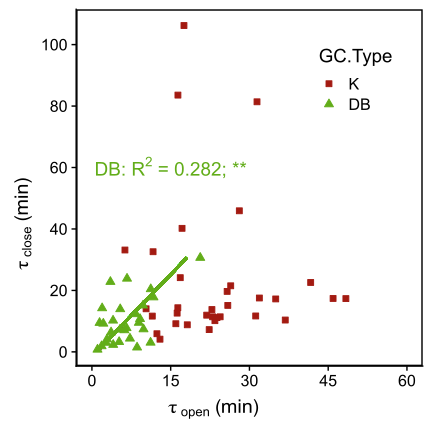


***Figure S5. Relationship between the time constants for opening (τ_open_) and closing (τ_close_) as per SMA-regression.*** Red squares indicate kidney-shaped stomata (K), green triangles indicate dumbbell-shaped stomata (DB). Stars indicate the significance level of the regression; ** > 0.01.


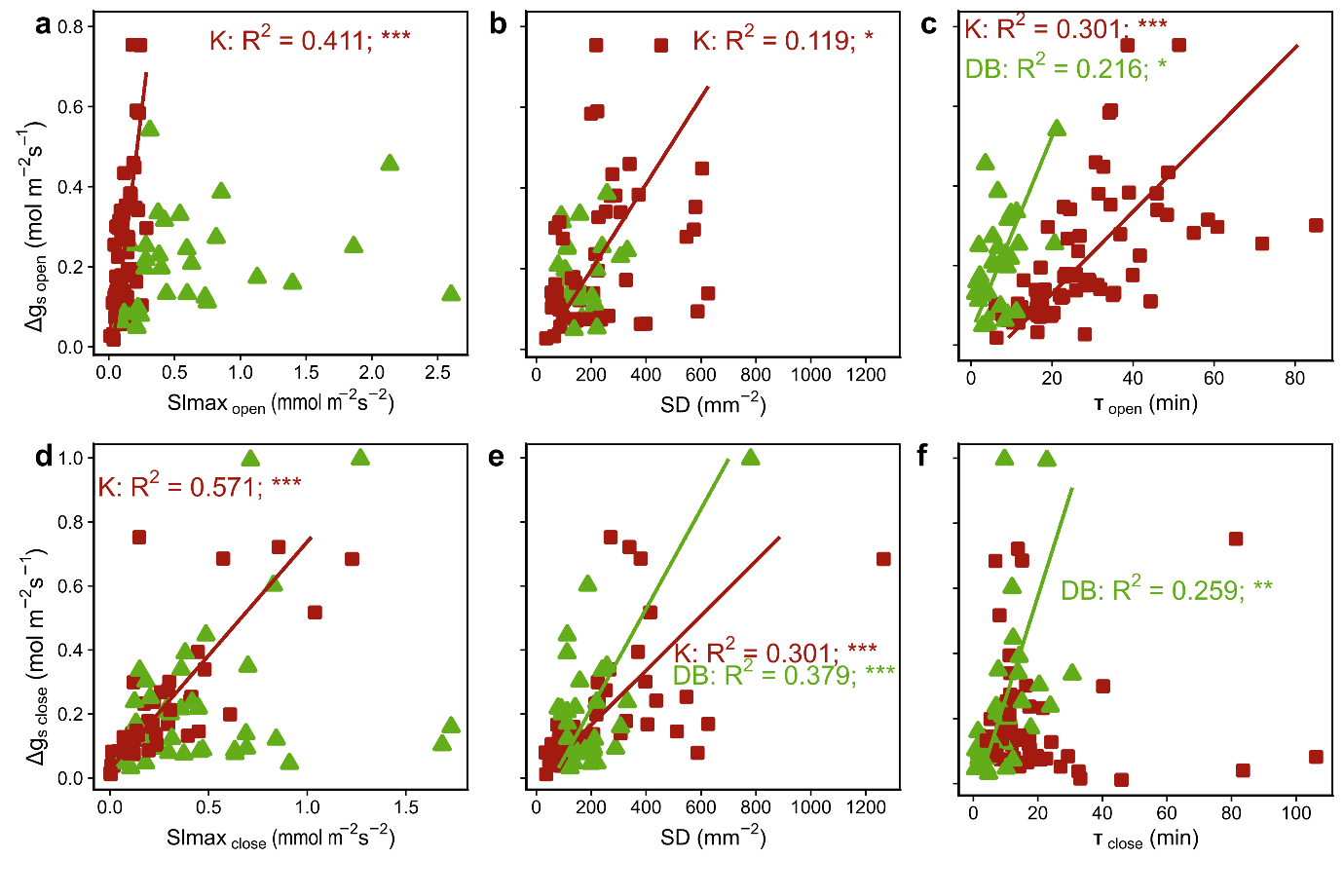
 ***Figure S6. Relationships between the difference in initial and final steady state stomatal conductance*** during opening (*Δg_s open_*) and **(a)** maximum speed of opening (*Slmax_open_*), **(b)** stomatal density (*SD*), and **(c)** the time constant of opening (*τ_open_*). Relationships between the difference in initial and final steady state conductance during closing (*Δg_s close_*) and **(a)** maximum speed of closing (*Slmax_close_*), **(b)** *SD*, and **(c)** time constant of closing (*τ_close_*; min). Red squares indicate kidney-shaped stomata (K), green triangles indicate dumbbell-shaped stomata (DB). Regression lines are added when significant relationships between variables were found as per SMA-regression. Stars indicate the significance level of the regression; * > 0.05, ** > 0.01, *** > 0.001.


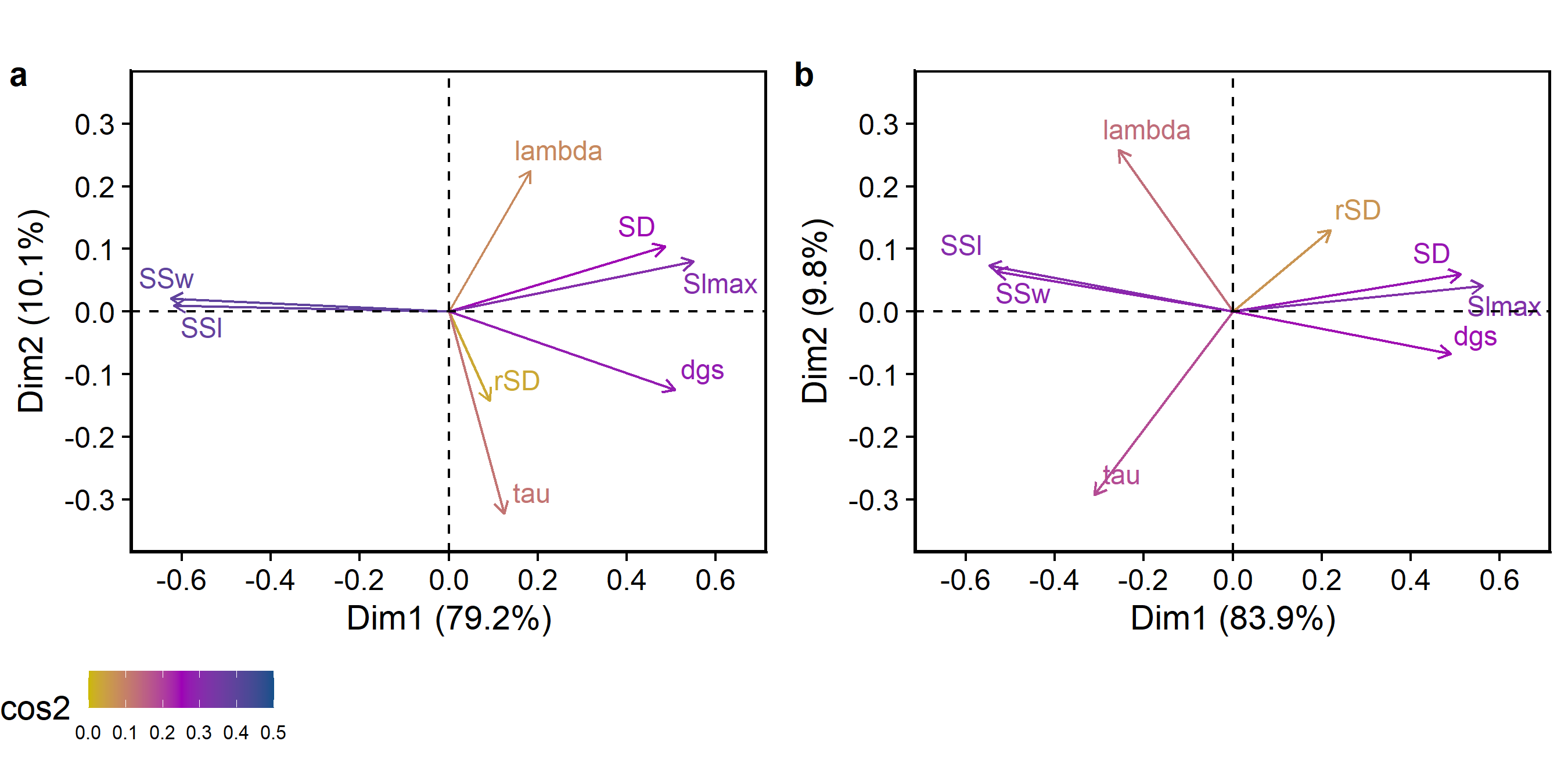
 ***Figure S7. Principal Component Analysis*** for **(a)** stomatal opening and **(b)** stomatal closing in kidney-shaped stomata. The percentage on the axis shows the contribution of each principal dimension to the total variation. The color shows the contribution strength of each component to the dimensions, while the direction shows to which dimension it contributes. Components pointing in the same direction are positively correlated, components pointing in opposite directions are negatively correlated. When two component directions are offset by 90°, they are considered unrelated. *SD* refers to stomatal density, *SSl* refers to stomatal length, *rSD* refers to the ratio of adaxial/abaxial *SD*, *tau* refers to the time-constant, *lambda* to the lag constant, *dgs* to the magnitude in change of stomatal conductance, and *Slmax* to the maximum slope of the change in stomatal conductance.


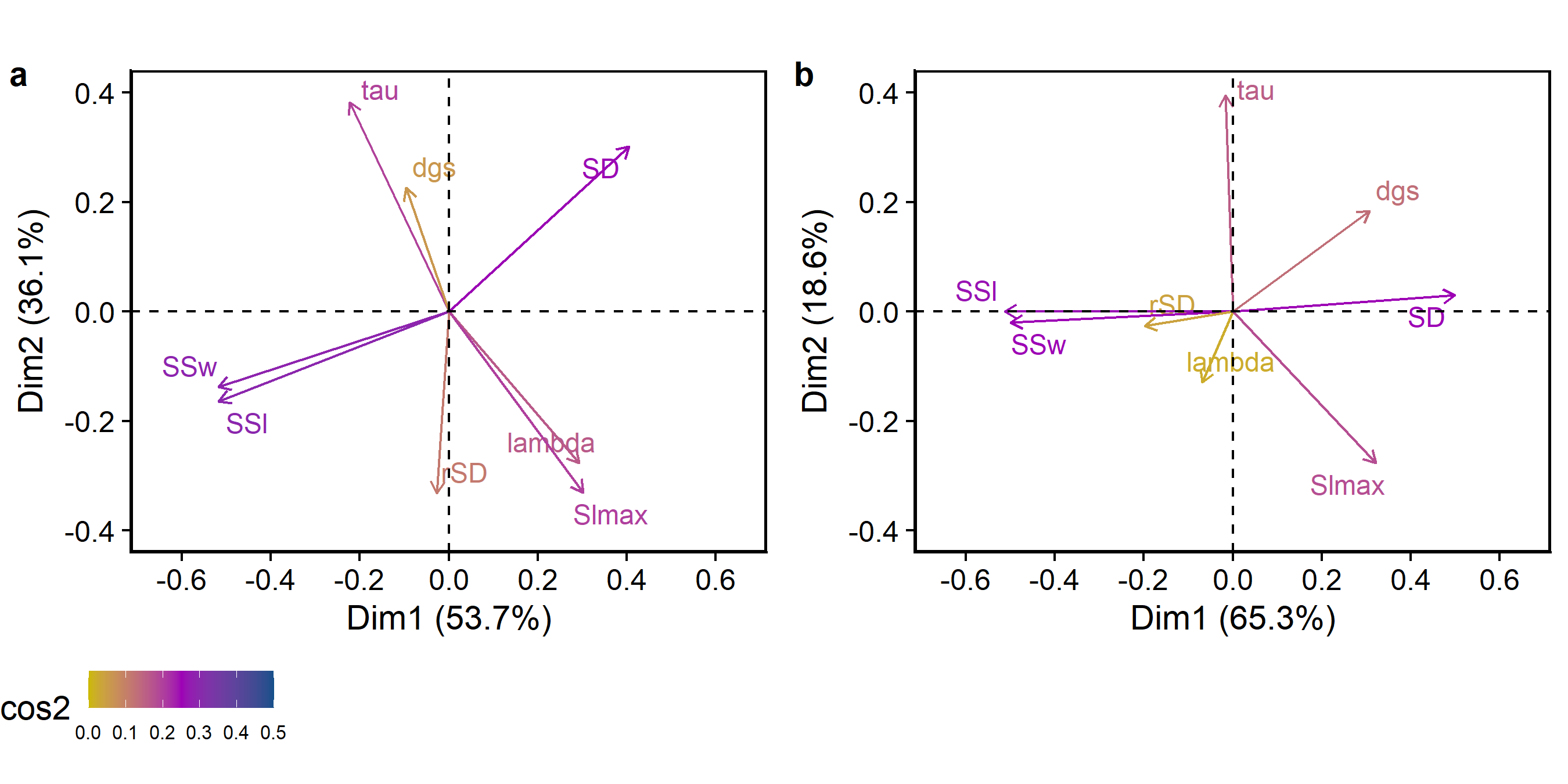
 ***Figure S8. Principal Component Analysis*** for **(a)** stomatal opening and **(b)** stomatal closing in dumbell-shaped stomata. The percentage on the axis shows the contribution of each principal dimension to the total variation. The colour shows the contribution strength of each component to the dimensions, while the direction shows to which dimension it contributes. Components pointing in the same direction are positively correlated, components pointing in opposite directions are negatively correlated. When two component directions are offset by 90°, they are considered unrelated. *SD* refers to stomatal density, *SSl* refers to stomatal length, *rSD* refers to the ratio of adaxial/abaxial *SD*, *tau* refers to the time-constant, *lambda* to the lag constant, *dgs* to the magnitude in change of stomatal conductance, and *Slmax* to the maximum slope of the change in stomatal conductance.


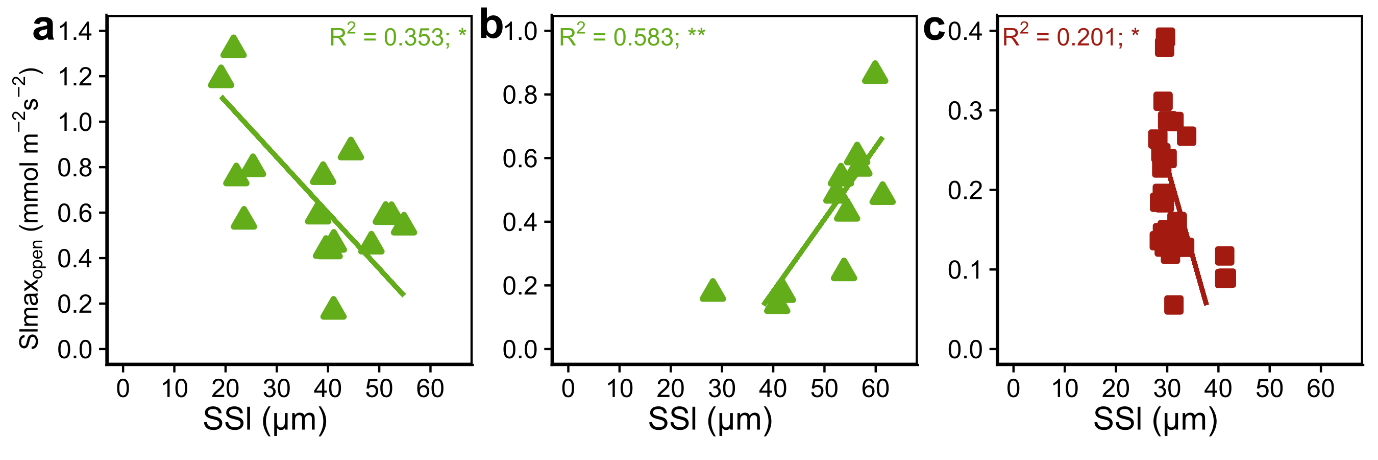


***Figure S9. Relationships between stomatal length (SSl) and maximum speed of stomatal opening (Slmax_open_)*** for (**a**) *Hordeum vulgare* (16 single-replicate datasets; 2 publications), (**b**) *Triticum aestivum* (11 single-replicate datasets; 2 publications) and (**c**) *Solanum lycopersicum* (26 single-replicate datasets; 2 publications). Contrary to the analysis in the rest of this work, this analysis was performed on paired observations of single replicates, not per ‘set’. This is thus a similar analysis as performed in earlier published works on correlations between stomatal speed and size. Stars indicate the significance level of the found regression; * > 0.05, ** > 0.01, *** > 0.001 as per SMA-regression.

**REFERENCES:**

**McAusland L, Vialet-Chabrand S, Davey P, Baker NR, Brendel O, Lawson T**. **2016**. Effects of kinetics of light-induced stomatal responses on photosynthesis and water-use efficiency. *New Phytologist* **211**: 1209–1220.

**Ozeki K, Miyazawa Y, Sugiura D**. **2022**. Rapid stomatal closure contributes to higher water use efficiency in major C4 compared to C3 Poaceae crops. *Plant Physiology* **189**: 188–203.
